# Hepatocyte serine palmitoyltransferase 3 is essential for mitochondrial ceramide homeostasis and electron transport chain function

**DOI:** 10.1016/j.jbc.2026.113630

**Published:** 2026-10-09

**Authors:** Maryam Jamil, Anna Kovilakath, Yolander Valentine, Saaid Farooq, David Montefusco, Yang Yue, Gisela Beutner, Qun Chen, Jeremy Thompson, Komuraiah Myakala, Xiaoxin Wang, Christiane Carter, Jinze Liu, Arjun Rijal, Charles E. Lyons, Devanand Sarkar, Moshe Levi, George A. Porter, Sushil Mahata, Tytus Bernas, Edward J. Lesnefsky, L. Ashley Cowart

**Affiliations:** 1 Department of Cellular, Molecular, and Genetic Medicine, Virginia Commonwealth University, Richmond, Virginia, USA; 2 Division of Cardiology, Department of Internal Medicine, Pauley Heart Center, Virginia Commonwealth University, Richmond, Virginia, USA; 3 C. Kenneth and Dianne Wright Center for Clinical and Translational Research, Virginia Commonwealth University, Richmond, Virginia, USA; 4 Department of Pediatrics, University of Rochester Medical Center, Rochester, New York, USA; 5 Department of Biochemistry and Molecular & Cellular Biology, Georgetown University, Washington, District of Columbia, USA; 6 Department of Biostatistics, School of Medicine, Virginia Commonwealth University, Richmond, Virginia, USA; 7 Massey Comprehensive Cancer Center, Virginia Commonwealth University, Richmond, Virginia, USA; 8 Department of Pharmacology and Physiology, University of Rochester Medical Center, Rochester, New York, USA; 9 Aab Cardiovascular Research Institute, University of Rochester Medical Center, Rochester, New York, USA; 10 VA San Diego Healthcare System and University of California San Diego, California, USA; 11 Department of Anatomy and Neurobiology, Virginia Commonwealth University, Richmond, Virginia, USA; 12 Richmond Veterans’ Affairs Medical Center, Richmond, Virginia, USA

**Keywords:** SPTLC3, ceramides, complex I, ETC, hepatocytes

## Abstract

Sphingolipids (SLs) constitute essential membrane lipids that also function as signaling molecules mediating fundamental cellular processes. Disruption of constitutive SL metabolism contributes to a range of metabolic diseases. SL biosynthesis is initiated by serine palmitoyltransferase (SPT), a heteromeric enzyme composed of Sptlc1 and either Sptlc2 or the less-characterized subunit Sptlc3. While the canonical Sptlc1/Sptlc2 complex generates most SLs, Sptlc1/Sptlc3 produces noncanonical SLs that remain poorly understood. Sptlc3 expression increases in liver disease in both mouse models and humans, yet its biological role remains unclear. To address this, we generated a liver-specific SPTLC3 knockout mouse model. Loss of SPTLC3 resulted in severe mitochondrial dysfunction in hepatocytes, characterized by reduced oxygen consumption, decreased ATP production, and elevated NADH/NAD^+^ ratios with a concomitant increase in glycolytic activity. Mechanistically, we found a defect in the electron transport chain (ETC) which stemmed not from intrinsic impairment of the ETC complexes but rather from hindered electron flow between complex I and complex III likely due to reduced availability of Coenzyme Q. While atypical ceramides constituted (∼4% of total ceramides) when measured in liver homogenates, there measurements in mitochondria-enriched fractions revealed enrichment in this cell compartment. Together, our findings identify SPTLC3 as a critical modulator of mitochondrial ceramide composition and ETC function, revealing a specialized role for noncanonical SLs in hepatocyte energy metabolism.

Sphingolipids (SLs) constitute a class of bioactive lipids forming structural components of cellular and organelle membranes and mediating multiple cellular processes across eukaryotic cell types (1). The initial step in SL biosynthesis occurs through the action of serine palmitoyltransferase (SPT), which condenses an amino acid with a fatty acyl-CoA. Two subunits make up the catalytic core of SPT: Sptlc1, which dimerizes with either Sptlc2 or the more recently discovered Sptlc3 which generates the initial SL in the pathway, the long-chain base (LCB) 2, 3, 4. This LCB serves as the scaffold upon which all downstream SLs are synthesized, including ceramides, sphingomyelin, glycolipids, and sphingosine-1-phosphate. The SPT enzyme complex formed by Sptlc1 and Sptlc2 is highly specific for serine and palmitoyl-CoA, thereby synthesizing LCBs with an 18-carbon backbone (16 carbons from palmitate and 2 from serine). In contrast, while the more recently described SPT heterodimer of Sptlc1 and Sptlc3 also generates d18:0 bases, it exhibits a broader range of acyl-CoA utilization, thereby generating LCBs spanning from C16 to C20, though the significance of these atypical lipids remains poorly understood (3).

The liver is comprised of multiple cell types including hepatocytes, hepatic stellate cells, Kupffer cells, epithelial cells, and macrophages. Notably, hepatocytes, constituting 70 to 80% of liver mass 5, 6, carry a significant metabolic load. Central to the hepatocyte’s metabolic orchestration are mitochondria, the metabolic powerhouses governing glucose, lipid, and protein metabolism, thereby ensuring cellular energy production and overall cellular and organismal homeostasis. Hepatocytes contain more mitochondrial mass than other liver cell types (7), and subsequently, mitochondria constitute approximately 13 to 20% of the liver volume, and mitochondrial dysfunction manifests in various manners including elevated levels of reactive oxygen species, damage to mitochondrial DNA (mtDNA), disturbances in ATP synthesis, alterations in mitochondrial morphology and structure, dysregulation of calcium homeostasis, abnormal mitophagy, and the potential activation of programmed cell death 8, 9, 10. Subsequently, they play a crucial role in the occurrence and progression of various liver diseases 11, 12.

SLs including ceramides, sphingomyelins, and gangliosides are constituents of the mitochondrial membrane (13). Ceramide and S1P in particular, regulate mitochondrial function influencing mitochondrial dynamics, bioenergetics, cell death pathways, and morphology 14, 15, 16, 17. SLs have been implicated in multiple liver disorders as well as other aspects of liver pathology, such as diabetes, obesity, insulin resistance, and others 12, 18, 19, 20, 21, 22, 23, 24. Though little is known regarding the biological functions of Sptlc3 and its atypical SL products, multiple studies have reported SPTLC3 upregulation in the progression of nonalcoholic steatohepatitis to hepatocellular carcinoma (HCC) and nonalcoholic fatty liver disease or otherwise implicated SPTLC3 and its products in liver disease 25, 26, 27. Moreover, we recently demonstrated that Sptlc3-derived sphingomyelin modulates adenylate cyclase activity at the plasma membrane in hepatocytes (28).

To investigate the role of Sptlc3 and its products in hepatocyte SL metabolism, we generated a hepatocyte-specific SPTLC3 knockout (SPT3-hKO) mouse model (28). Our prior work established that SPTLC3 is critical for maintaining mitochondrial morphology and function in cardiomyocytes, where its loss led to impaired complex I activity of the electron transport chain (ETC) (29). Importantly, in that study we identified Sptlc3-derived lactosylceramides in direct contact with complex I, which led to a fundamental defect in NADH oxidoreductase activity, the major function of the complex. In contrast, the current study reveals a hepatocyte-specific role for SPTLC3 in mitochondrial function independent of a fundamental enzymatic defect. Nonetheless, similar to that study, depletion of Sptlc3 in hepatocytes impaired mitochondrial function, SL content, morphology, and ATP production. Mechanistically, loss of Sptlc3 disrupted the mitochondrial lipid composition, not just with respect to SLs but more broadly, and impaired electron transfer from complex I to complex III of the ETC, which in turned compromised mitochondrial respiration and overall cell energy homeostasis. Collectively, our findings highlight the tissue-specific role of SPTLC3 in regulating mitochondrial function and underscore its unique contribution to hepatocyte bioenergetics.

## Results

### Depletion of SPTLC3 compromises mitochondrial function and ultrastructure

The SPTLC3 floxed mouse model was generated by our group as previously described 28, 29 (Fig. S1, *A* and *B*). Immunoblotting for Sptlc3 in primary hepatocytes harvested from control and SPT3-hKO mice, demonstrated very low levels of Sptlc3, confirming effective depletion (Fig. 1*A*). Whereas no change was observed in expression of SPTLC1 and SPTLC2 (Fig. S1*C*). To determine the relative distribution of SPTLC2 and SPTLC3 within SPT complexes, we performed SPTLC1 coimmunoprecipitation followed by mass spectrometry-based quantification in HC3716 hepatocytes. Neither SPTLC1, SPTLC2, nor SPTLC3 was detected in IgG control precipitates, confirming assay specificity, whereas SPTLC1 (the bait) was recovered at high abundance across all replicates. Both SPTLC2 and SPTLC3 were reproducibly coimmunoprecipitated with SPTLC1; however, SPTLC2 abundance was consistently higher than that of SPTLC3 across replicates, indicating that SPTLC2 is the predominant SPTLC1-binding partner in these cells, while a smaller but reproducible pool of SPTLC1/SPTLC3 complexes was also detected (Fig. S1*D*).

**Figure 1:**
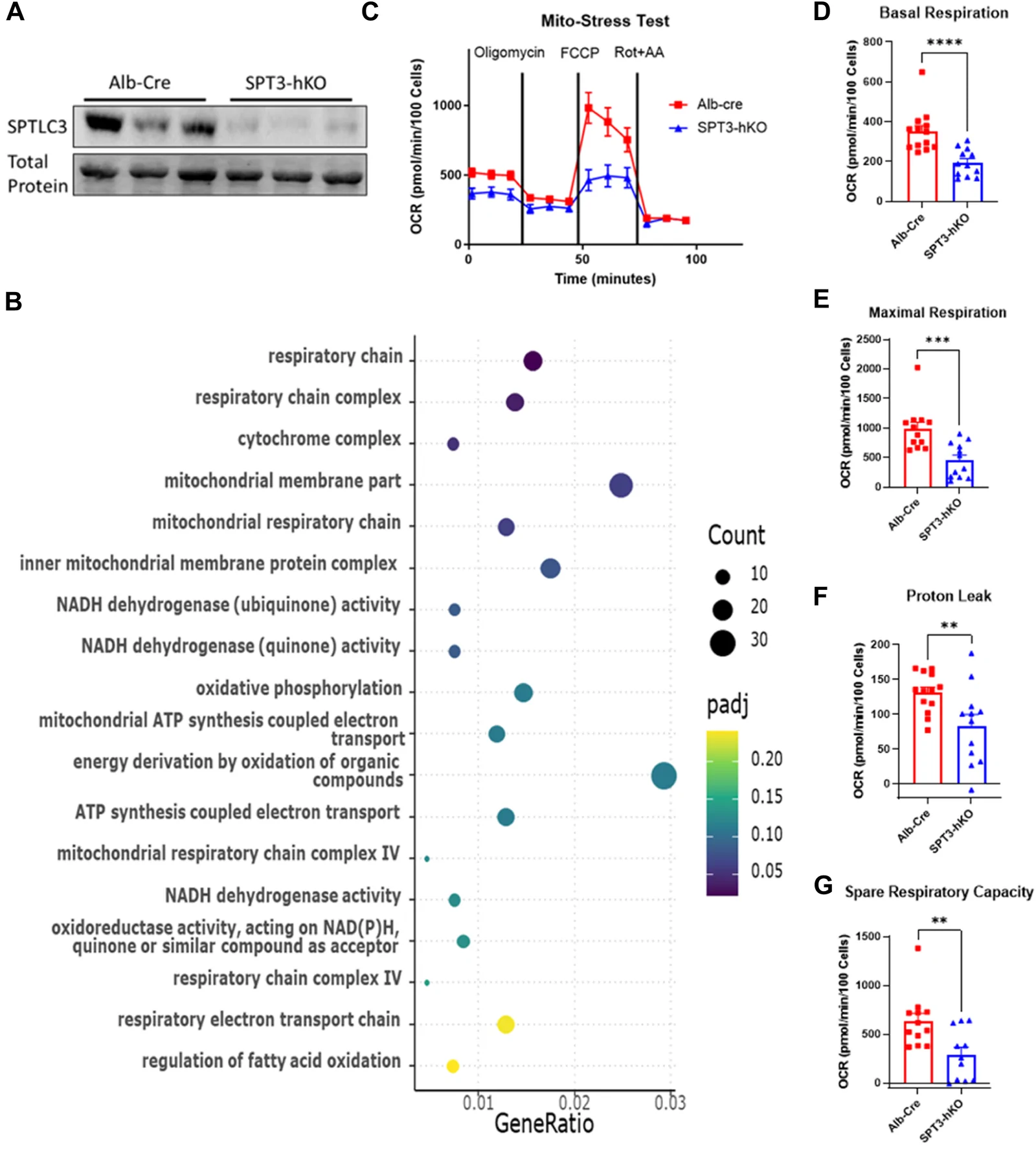

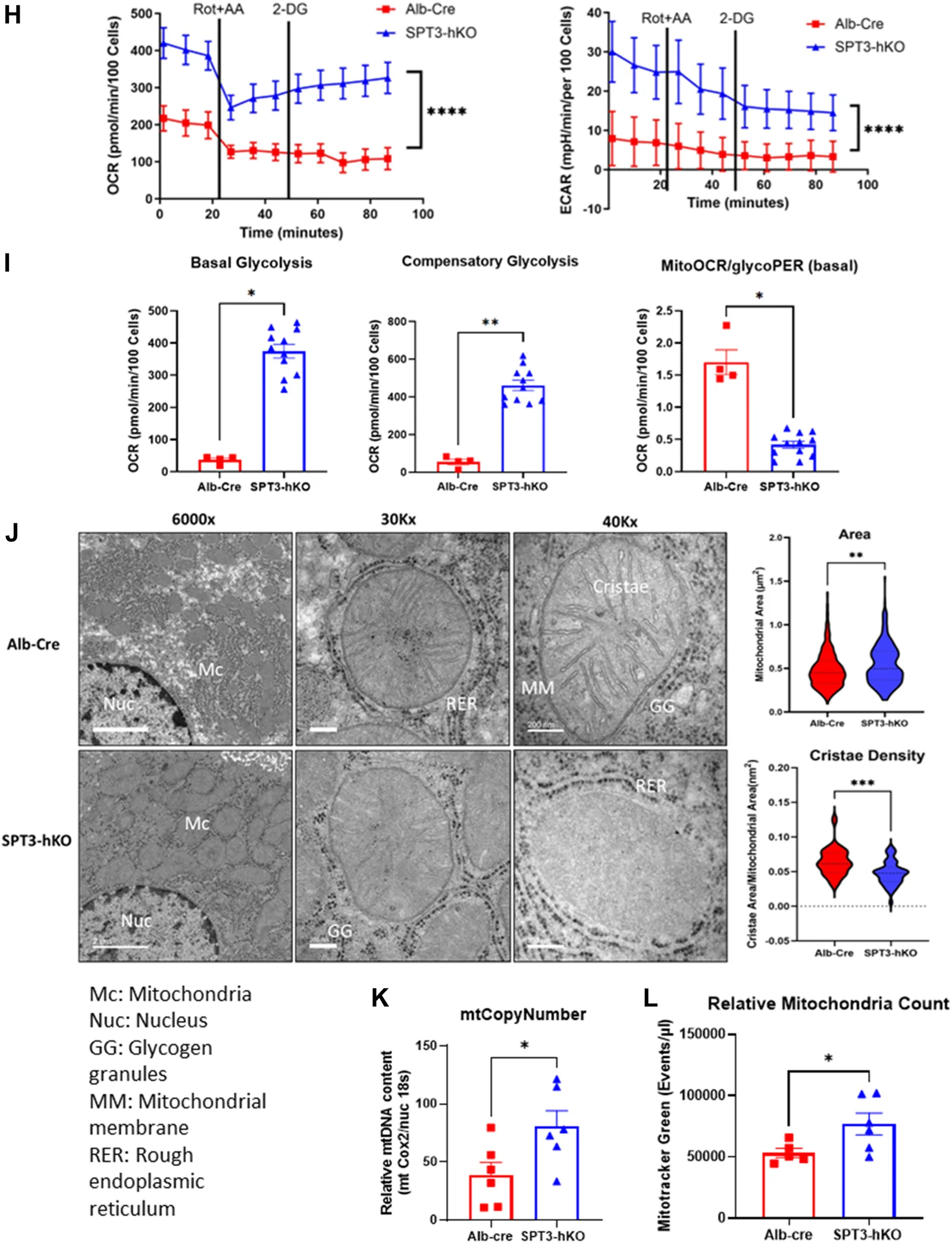
**SPTLC3 deletion induces mitochondrial dysfunction in murine hepatocytes.***A*, Immunoblotting shows the protein expression of Sptlc3 along with total protein in primary hepatocyte isolates from Alb-cre controls and SPT3-hKO mice (N = 3/group). *B*, Gene Ontology overrepresentation analysis (Fisher's exact test) of differentially expressed genes identified through RNA-seq analysis (alb-cre, N = 3; SPT3-hKO, N = 5). *C*–*G*, Mito-stress test conducted using the seahorse assay on primary hepatocytes from alb-cre and SPT3-hKO mice, measuring oxygen consumption rate (OCR) per 100 cells (N = 12/group). *H* and *I*, Glycolytic rate test performed using the seahorse assay on primary hepatocytes from alb-cre and SPT3-hKO mice, with OCR and ECAR normalized per 100 cells (N = 3/group). *J*, representative transmission electron microscopy (TEM) images of mitochondria obtained from liver sections at 6000 x, 30 Kx, and 40 Kx magnification in alb-cre and SPT3-hKO mice. Quantitative analysis conducted on more than 300 mitochondria per group to calculate mitochondrial area and on 25 to 45 images per group to assess cristae density (N = 2/group). Analysis performed using the freehand tool in ImageJ. *K*, mitochondrial DNA copy number calculated by quantitative PCR (qPCR) analysis of isolated DNA from primary hepatocytes (N = 3). *L*, relative mitochondrial count determined by flow cytometry on isolated mitochondria stained with MitoTracker *Green* (N = 6/group). The data presented are means ± SEM. Statistical analysis performed using unpaired Student's *t* test. ∗*p* < 0.05, ∗∗*p* < 0.01, ∗∗∗*p* < 0.001, ∗∗∗∗*p* < 0.0001. SPT3-hKO, SPTLC3 knockout.

To investigate the role of SPTLC3 in liver biology, RNA-seq analysis was conducted on liver homogenates from adult male control (Alb-Cre^+^) and SPT3-hKO mice. Building on our previous findings from the cardiomyocyte-specific SPT3-hKO model (29), we focused on pathways related to mitochondrial function. This analysis revealed the most significant differences in Gene Ontology (GO) pathways associated with the respiratory chain complex, mitochondrial membranes, and other aspects of mitochondrial metabolism, particularly with respect to the ETC and oxidative phosphorylation (Fig. 1*B*). To further assess the impact of Sptlc3 depletion on mitochondrial function, Seahorse respirometry was utilized to measure oxygen consumption rates (OCRs) in primary hepatocytes isolated from control *versus* SPT3-hKO mice. Depletion of Sptlc3 notably decreased the OCR in several aspects of mitochondrial function including basal respiration, maximal respiration, ATP production, and proton leak (Fig. 1, *C–H*). The most significant difference was observed in uncoupled maximal respiration, where the electron flow through the ETC is uninhibited and complex IV reaches maximum capacity; therefore, our data suggested a potential ETC defect. Consistent with a decreased reliance on mitochondria for energy production, both basal and compensatory glycolytic rates were nearly 10-fold higher in SPT3-hKO hepatocytes, and similarly, the ratio of mitochondrial OCR to glycolytic rate was lower (Fig. 1*H*, right panel, and 1i). These data suggest that, in the absence of Sptlc3, hepatocyte metabolism shifts toward anaerobic glycolysis. Though this is not a preferred pathway of energy production for hepatocytes, it may be a compensatory mechanism of energy production upon compromise of mitochondrial metabolism. Together, these data indicate a requirement for Sptlc3 for mitochondrial oxidation and ATP production.

Because of the striking defect in hepatocyte energy metabolism in the SPT3-hKO hepatocytes, transmission electron microscopy (TEM) was performed on liver sections from control and SPT3-hKO mice to assess mitochondrial structure and morphology *in vivo*. Consistent with impaired mitochondrial metabolism, TEM imaging revealed typical signs of mitochondrial damage in SPT3-hKO mice, including damaged cristae. In fact, cristae were barely detectable at 400,00x magnification, despite clearly delineated cristae in control cells at this magnification (Fig. 1*J*). Furthermore, total mitochondrial area in EM images was significantly higher in SPT3-hKO livers, suggesting mitochondrial swelling, whereas cristae density was lower (Fig. 1*J*). Despite the decreased mitochondrial function, however, SPT3-hKO primary hepatocytes exhibited a higher mtDNA content (Fig. 1*K*), which was further supported by flow cytometry analysis of total mitochondrial count (Fig. 1*L*). Collectively, these observations indicate a defect in mitochondrial function and morphology, and this may suggest that increasing mitochondrial number may serve as a compensatory function. The observed mitochondrial abnormalities highlight the significance of Sptlc3 in hepatic energy metabolism and shed light on potential mechanisms underlying the liver pathology associated with Sptlc3 deficiency.

### Sptlc3 is essential for ETC

Because the decreased oxygen consumption suggested a potential defect in ETC, specific complexes of the ETC were assessed. Oroboros analysis of primary hepatocytes from SPT3-hKO and control mice revealed that the defect primarily arose from a deficiency in activity of complex I and Complex IV of the ETC (Fig. 2, *A–C*). While a combination of tricarboxylic acid (TCA) cycle substrates (pyruvate, malate, and glutamate) served as substrates in Oroboros analysis, the fundamental enzymatic activity of complex I is the oxidation of NADH produced in the TCA cycle to NAD+. Therefore, to distinguish between a TCA cycle defect or a defect in complex I, we directly assessed complex I function in enriched mitochondria from mouse livers. Since NADH cannot pass through the mitochondrial inner membrane in intact mitochondria, hindering its accessibility by the ETC, we employed freeze-thawed mitochondria to assess OCR directly after NADH addition, an approach used in previous studies (30). Seahorse was used for polarographic oxygen consumption assays for complex I (NADH), complex II (succinate), and complex IV (TMPD & ascorbate) activities 30, 31. Notably, the mitochondria derived from SPT3-hKO mice exhibited significantly lower oxygen consumption than controls when using NADH, supportive of a complex I defect, since complex I is the rate limiting step in this assay (Fig. 2, *D* and *E*). These mitochondria also showed reduced OCR following complex IV substrate addition, consistent with the Oroboros findings and indicating impaired complex IV activity, However, oxygen consumption in the presence of succinate—a complex II substrate—was comparable between genotypes suggesting that Complex II restores downstream activity by bypassing Complex I (Fig. 2, *F* and *G*). Overall, these data suggest that the primary ETC defect in SPT3-hKO mitochondria arises from impaired Complex I and Complex IV function.

**Figure 2:**
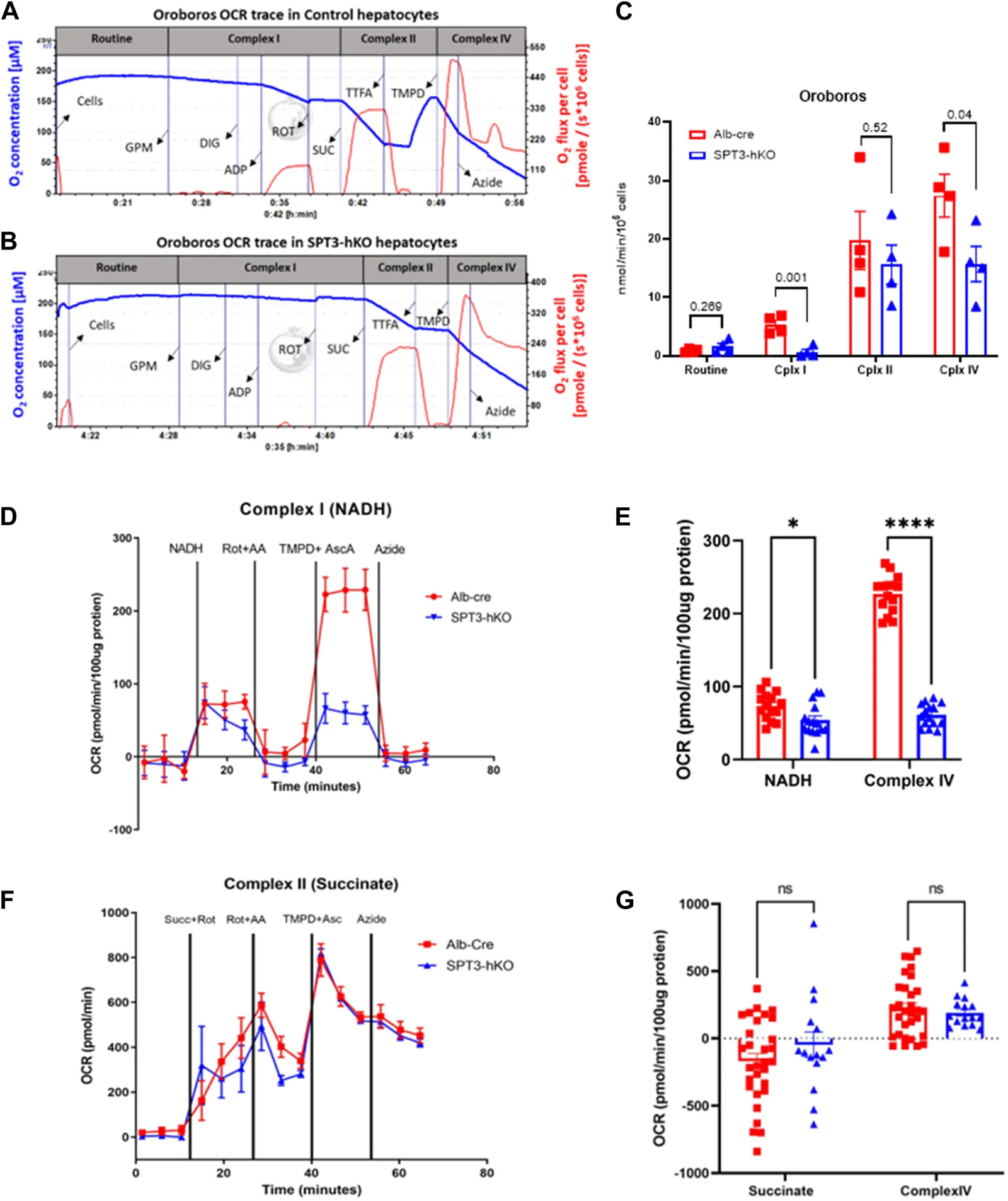

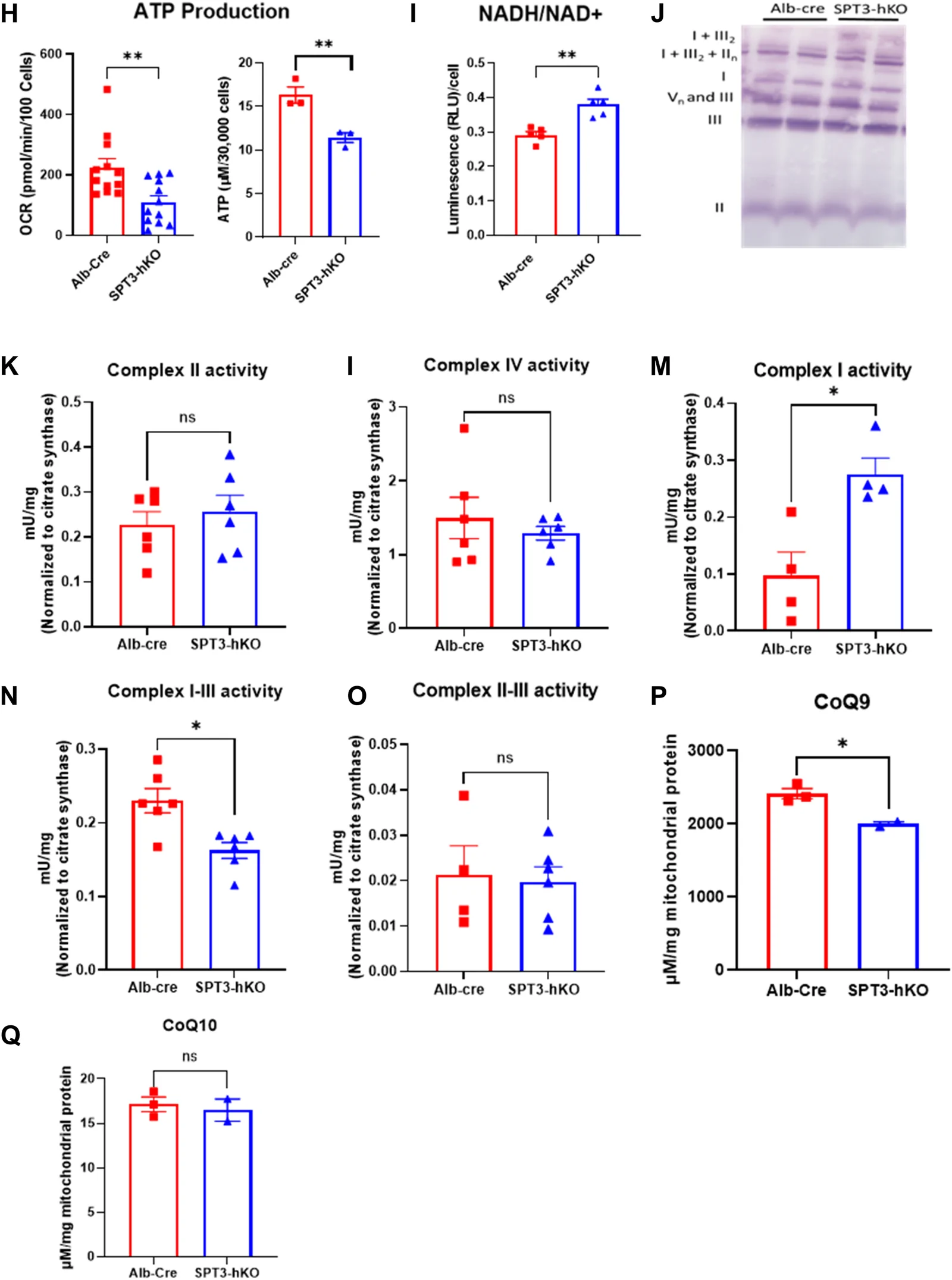
**Mitochondrial dysfunction in SPT3-hKO resulting from complex I defect in the electron transport chain (ETC).***A* and *B*, Oroboros O2K representative tracing of a. alb-cre (control) and b. SPT3-hKO primary hepatocytes using mitochondrial-specific complex protocol. *Arrows* represent addition of each injection with substrates/inhibitors/uncouplers, glutamate (GPM) 10 mM + pyruvate 5 mM + malate 2 mM, digitonin (DIG) 10 μg/ml, adenosine diphosphate (ADP) 2 mM, rotenone (ROT) 0.5 μM, succinate (SUC) 10 mM, thenoyltrifluoroacetone (TTFA) (40 μM), tetramethylphenylenediamine (TMPD) 0.3 mM + ascorbate 3 mM, and azide: 15 mM. *C*, quantification of Oroboros in primary hepatocytes after the addition of substrates for Complex I, II, and IV. OCR in nmol/min normalized to 10^6^ cells (N = 3/group). *D*–*G*, Seahorse analysis conducted on frozen-thawed mitochondria from alb-cre and SPT3-hKO livers to assess the response to specific ETC substrates: NADH (Complex I substrate), and Succinate (Complex II substrate). OCR normalized to 100 μg of protein (N = 4). *H*, ATP production calculated using seahorse assay and ATP production luminescence assay, normalized to cell count (N = 3). *I*, calculation of the NADH/NAD+ ratio using the NADH assay, normalized to cell count (N = 3). *J*, Blue Native page displaying the formation of native Complex I, II, III, V, and supercomplexes in mitochondria from alb-cre and SPT3-hKO (N = 3, representative image is shown). *K*–*O*, Evaluation of Complex II activity (succinate dehydrogenase), Complex IV activity (Cytochrome c-oxidoreductase), Complex I activity (NADH-ubiquinone oxidoreductase), Complex I-III activity (0.3 mM NADH + Cytochrome *c*), and Complex II-III activity (succinate-cytochrome *c* oxidoreductase) normalized to citrate synthase (N = 6). *P* and *Q*, quantification of Coenzyme Q (CoQ9, CoQ10) in mitochondria from alb-cre and SPT3-hKO livers (N = 3/group). The data shown represent means ± SEM. Statistical analysis was performed using unpaired Student's *t* test. ∗*p* < 0.05, ∗∗*p* < 0.01, ∗∗∗*p* < 0.001, ∗∗∗∗*p* < 0.0001. SPT3-hKO, SPTLC3 knockout; OCR, oxygen consumption rate.

Next, ATP and NADH levels were measured in primary hepatocytes. In line with our broader findings indicating a deficiency in complex I activity, SPT3-hKO hepatocytes demonstrated lower ATP concentrations than controls and a concomitant increase in the NADH/NAD+ ratio (Fig. 2, *H* and *I*), suggesting a defect in complexes I and IV and therefore ETC function. Furthermore, the ETC can form supercomplexes, wherein several individual complexes assemble, creating larger functional units that aid in electron transport. However, analysis of supercomplex formation and the natural expression of complex I through blue native PAGE showed no discernible distinctions between the SPT3-hKO and control samples (Fig. 2*J*).

While previous measurements focused on intact cells and enriched mitochondria, activity assays were conducted in detergent-solubilized membranes from mitochondria to investigate the activities of complex function *in vitro*. As expected, no differences were observed in complex II activity (Fig. 2*K*). Furthermore, complex IV activity showed no significant difference in control *versus* SPT3-KO (Fig. 2, *L* and *M*). A notable enhancement was observed in the detergent-solubilized complex I enzymatic activity (*i.e.*, conversion of NADH to NAD+) *in vitro*. This shows that with exogenous CoQ provided, complex I is fully functional. Nevertheless, complex I-III linked activity (NADH:cytochrome *c* oxidoreductase; NADH→endogenous coenzyme Q→complex III→exogenous cytochrome *c*) was significantly lower in SPT3-hKO mitochondrial samples (Fig. 2*N*) whereas complex II-III linked activity (succinate:cytochrome *c* oxidoreductase; succinate→endogenous coenzyme Q→complex III→exogenous cytochrome *c*) remained unchanged (Fig. 2*O*). Together, these findings suggest that while individual complexes retain full activity when supplied with exogenous CoQ, the electron transfer from complex I to III is substantially impeded indicating CoQ deficiency. Therefore, both CoQ9 and CoQ10 were assessed in mitochondria from both control and SPT3-hKO mice. CoQ9, the most prevalent murine ubiquinone and abundant in liver (32), was significantly lower in SPT3-hKO mitochondria, while CoQ10 showed no significant difference (Fig. 2, *P* and *Q*). These data suggest that the decline in complex I function may arise, at least in part, from the reduced availability of CoQ9.

### SPTLC3 knockdown in human hepatic cells recapitulates mitochondrial defects observed in isolated mouse hepatocytes

To assess whether the effects of Sptlc3 deficiency observed in mice can be replicated in human cells, we employed siRNA knockdown SPTLC3 in immortalized human hepatic cells (HC-3716). Knock down of SPTLC2, the canonical Sptlc1 partner, was also used to test whether these functions are specific to Sptlc3 and atypical sphinogolipids. The efficiency of knockdown was confirmed through Western blot analysis (Fig. 3*A*). Similar to observations in the murine system, SPTLC3 knockdown resulted in a significant reduction in OCR, specifically affecting basal respiration, maximal respiration, ATP production, and spare respiratory capacity (Fig. 3, *B* and *C*). In contrast, loss of SPTLC2 did not significantly alter these parameters. Two different SPTLC3 siRNA were tested and produced same results (Fig. S2).

**Figure 3:**
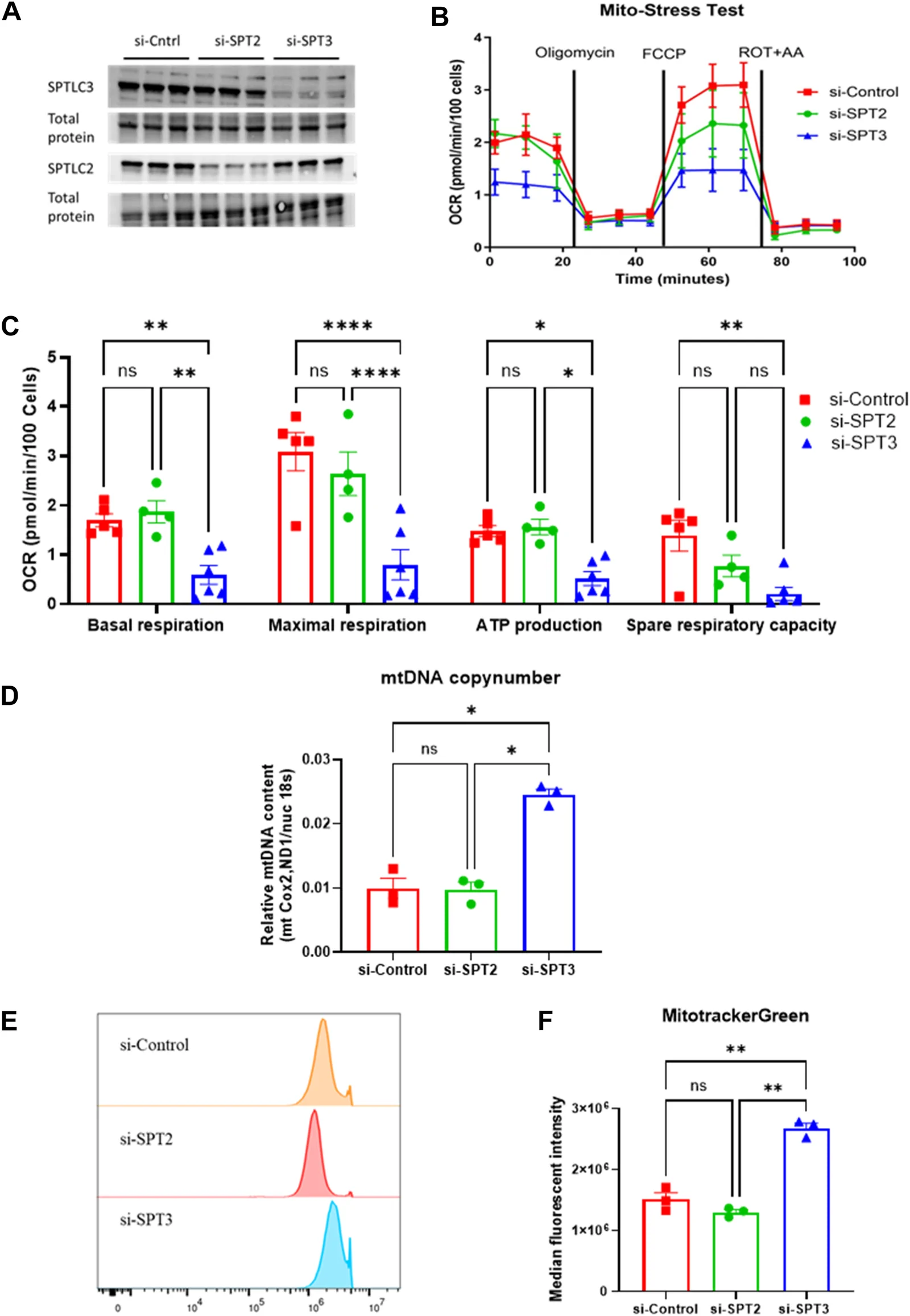

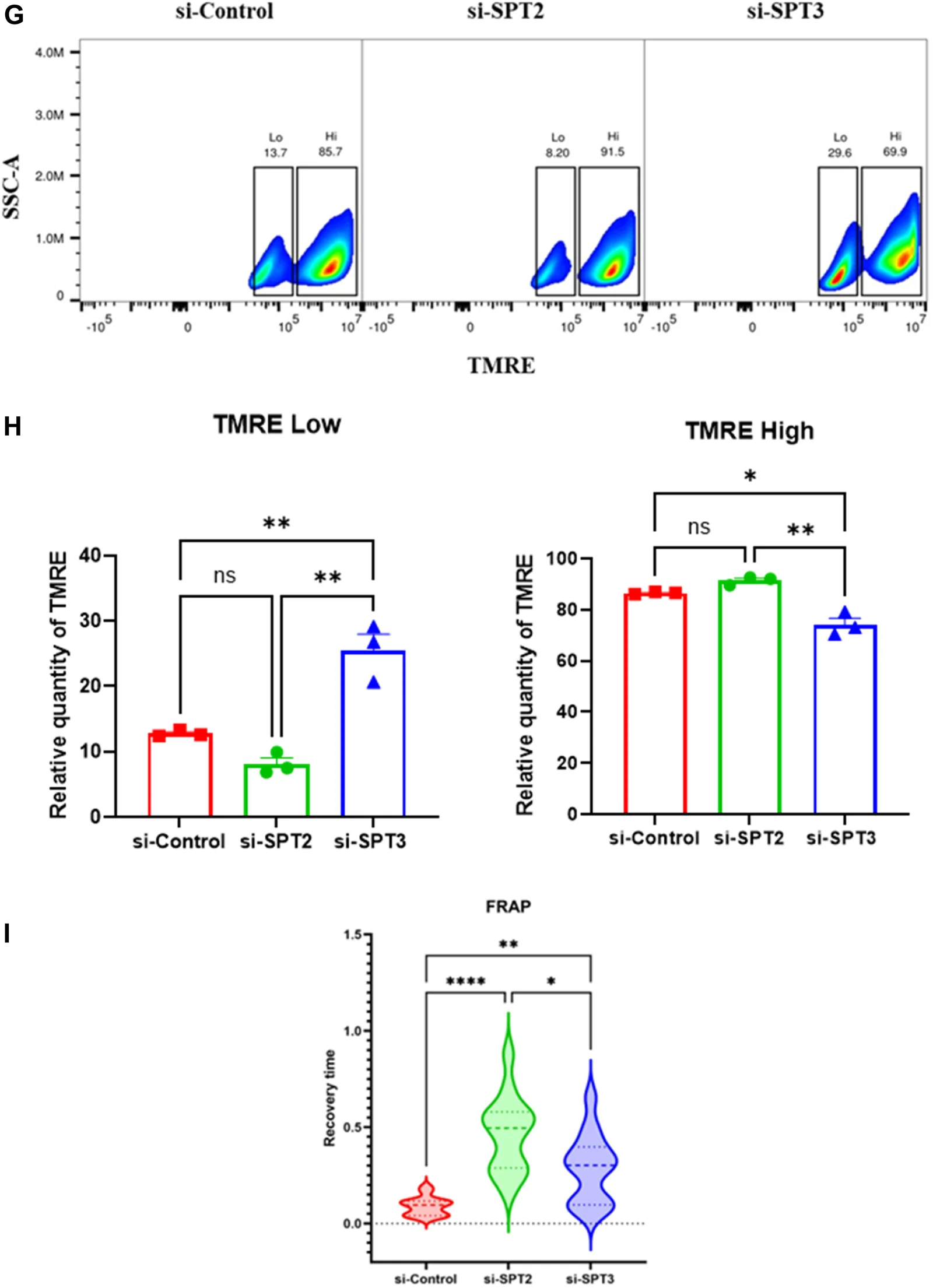
**Replication of murine findings through knockdown of SPTLC3 in human hepatic cell line.***A*, successful knockdown of SPTLC3 and SPTLC2 confirmed in human hepatic cell line HC3716 *via* Western blot analysis (N = 3/group). *B* and *C*, Mitostress test conducted on HC3716 cells after knockdown of SPTLC3 and SPTLC2 using seahorse assay followed by quantification of basal respiration, maximal respiration, ATP production, and spare respiratory capacity (N = 4–7/group). *D*, quantitative PCR analysis of mitochondrial DNA isolated from HC3716 cells after knockdown of SPTLC2 and SPTLC3 (N = 4). *E* and *F*, representative flow cytometry graph depicting median fluorescent intensity of Mitotracker *Green*; depicting mtDNA copy number; in HC3716 cells after knockdown of SPTLC2 and SPTLC3. Histogram represents the mean of three experiments. *G*, representative image depicting mitochondrial membrane potential in each group as measured by flow cytometry after TMRE staining. *H*, quantification of median fluorescent intensity reflecting low TMRE signal (N = 3/group). *I*, fluorescence recovery after photobleaching (FRAP) analysis conducted on HC3716 cells with knockdown of SPTLC2 and SPTLC3, showing the recovery of fluorescence in mitochondrial sections (5 sections each from 8 cells/group). The data presented are means ± SEM. Statistical analysis was performed using two way ANOVA. ∗*p* < 0.05, ∗∗*p* < 0.01, ∗∗∗*p* < 0.001, ∗∗∗∗*p* < 0.0001. SPT3-hKO, SPTLC3 knockout; TMRE, tetramethylrhodamine.

In addition to mitochondrial ETC function, mtDNA content and polarization were assessed. As with mouse hepatocytes, both mtDNA and MitoTracker Green staining were significantly increased in cells with SPTLC3 knockdown compared to the control indicating an increase in overall mitochondrial content (Fig. 3, *D*–*F*). Moreover, and consistent with the Mito Stress Test results, SPTLC2 knockdown did not affect DNA copy or polarization. To measure mitochondrial membrane potential, live cells were treated with tetramethylrhodamine (TMRE) to label active mitochondria. Intriguingly, the knockdown of SPTLC3 decreased the TMRE signal in the mitochondrial population, indicating an overall decline in mitochondrial membrane potential within SPTLC3-deficient cells (Fig. 3, *G* and *H*). Again, loss of Sptlc2 had no effect, indicating that Sptlc3 is specific for maintaining mitochondrial potential. Moreover, because knockdown of SPTLC3 in human hepatic cells reproduced the observed mitochondrial defects seen in mice, this system enabled demonstration that Sptlc2 is not critical for any of these mitochondrial functions/parameters, and these are specific molecular functions for Sptlc3-derived SLs.

SLs are generally hydrophobic and thus largely localized to cell and organelle membranes, where they influence membrane dynamics. Given than ETC complexes undergo conformational changes during respiration, we hypothesized that Sptlc3-dependent alternation in lipid composition may regulate the inner mitochondrial membrane (IMM) fluidity, where most ETC complexes reside. To test this, we performed fluorescence recovery after photobleaching (FRAP) analysis following either SPTLC2 or SPTLC3 knockdown (Fig. 3*I*). Cells were transfected with pDsRed2-Mito encoding a fusion of *Discosoma* sp. red fluorescent protein (DsRed2) and a mitochondrial targeting sequence of human cytochrome *c* oxidase subunit VIII (an IMM protein). Interestingly, both SPTLC2 and SPTLC3 knockdown induced a notable increase in recovery time, indicating that, while Sptlc3 is specific for maintenance of mitochondrial function, SLs in general (not specifically the atypical lipids synthesized *via* Sptlc1/3) appear to regulate membrane fluidity in the IMM. This is unsurprising, as numerous studies demonstrate functions for Sptlc2 in mitochondria. However, because depletion of only Sptlc3 but not Sptlc2 caused an ETC defect, it is unlikely that modified membrane fluidity is the primary cause of the mitochondrial deficiencies associated specifically with Sptlc3 deficiency.

### Sptlc3 depletion specifically impacts mitochondrial ceramides

To assess the actual impact of Sptlc3 on liver lipids, lipidomic analysis was performed on liver homogenates from control and SPT3hKO mice. Furthermore, because many defects observed in this study were observed in mitochondria, mitochondria were enriched from an aliquot of these liver homogenates. Western blot analysis confirmed mitochondrial enrichment in these fractions (Fig. 4*A*). As expected with differential centrifugation-based fractionation, these preparations also retained endoplasmic reticulum contamination, as indicated by residual Calreticulin signal in the mitochondria-enriched fraction (Fig. 4*A*). This ER contamination was comparable between genotypes, indicating that it does not account for the differences observed between groups. Untargeted LC-MS/MS performed on whole liver homogenates revealed partial separation between control and SPT3hKO groups in the PCA analysis, and no distinct clustering was observed in the heatmap of major lipid classes (Fig. 4, *B* and *C*). In contrast, lipidomics of the mitochondria showed a comparatively stronger separation between the genotypes in PCA analysis, along with clearly distinct clustering in the heatmap analysis (Fig. 4, *D* and *E*). Specifically, mitochondria from SPT3hKO livers exhibited significant alterations not only in SLs, such as sphingosines, but also in other lipid groups, mainly glycerolipids, glycerophosholipids, and triglycerides. Furthermore, while the mitochondrial cardiolipin profile remained similar between groups, di-lysocardiolipin were different between groups. These results suggest that deletion of SPTLC3 in hepatocytes disrupts overall lipid homeostasis in mitochondrial membranes (Table S1), particularly impacting structural membrane lipids and bioactive SLs, without broadly affecting overall hepatic membrane lipid composition.

**Figure 4:**
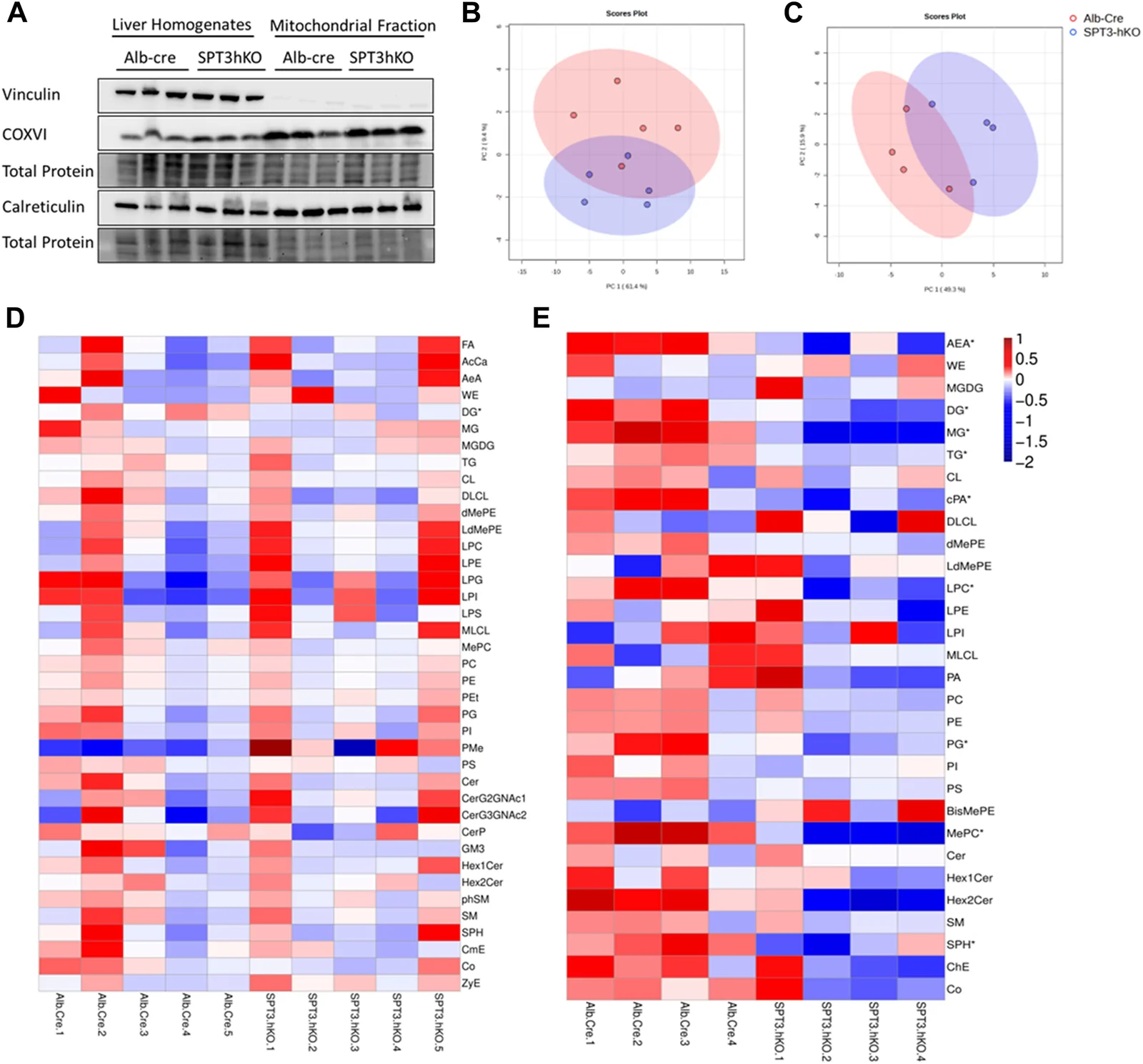

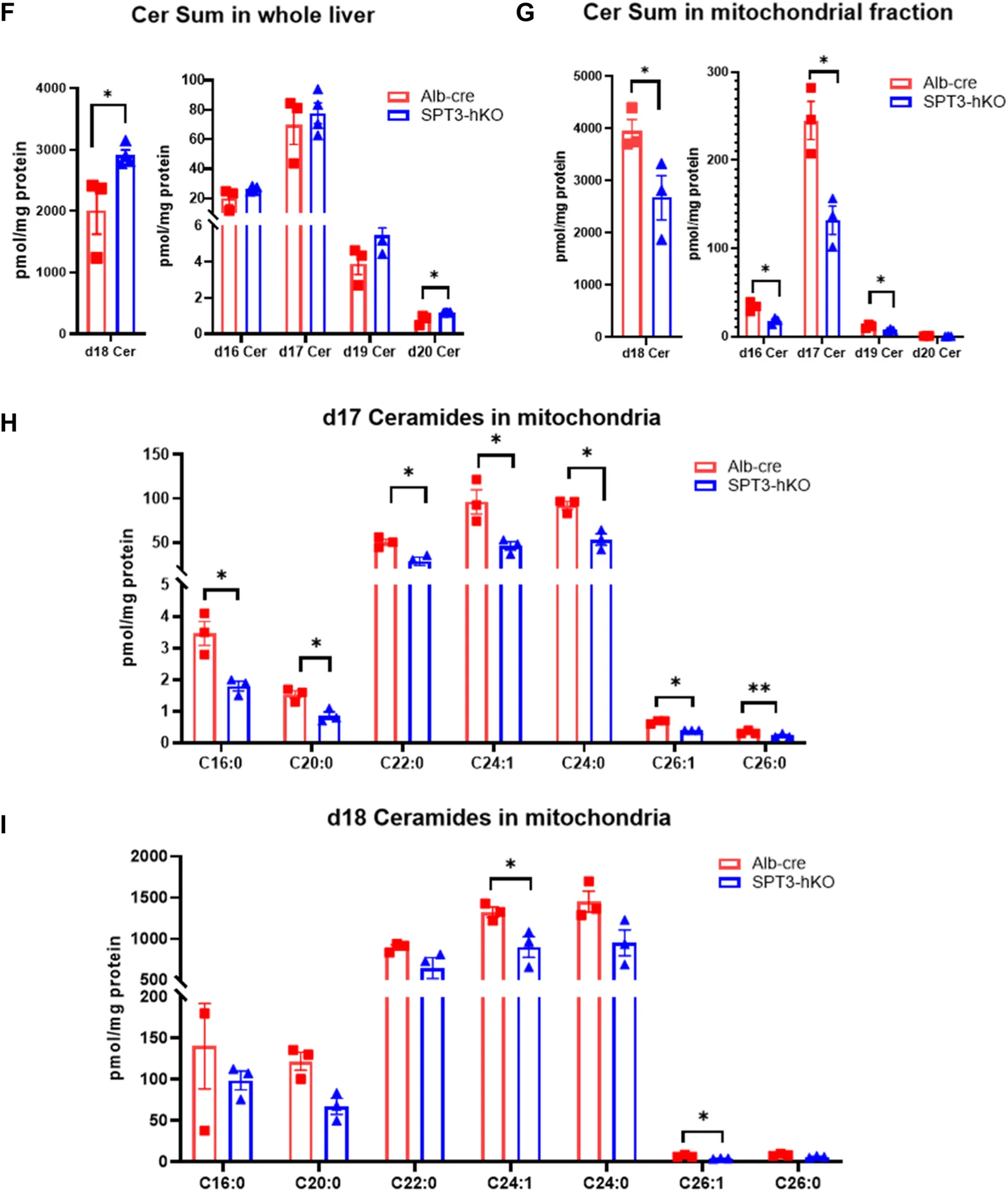
**Loss of SPTLC3 selectively alters mitochondrial ceramides.***A*, Western blot for vinculin (cytoplasmic marker), COXIV (mitochondrial marker), and calreticulin (ER marker) in liver homogenates and mitochondria-enriched fractions from livers of Alb-cre controls and SPT3-hKO mice (N = 3/group). *B*–*E*, untargeted lipidomics were performed on whole liver homogenates and enriched mitochondria from Alb-cre and SPT3-hKO mice (N = 5–6/group). Principal component analysis (PCA) score of lipid profiles in (*B*) whole liver and (*C*) enriched liver mitochondria. Hierarchical clustering heat maps of lipid species in (*D*) whole liver and (*E*) enriched liver mitochondria; *red* indicates upregulation, *blue* indicates downregulation. Columns represent individual samples; rows represent lipid species. *F*–*I*, targeted sphingolipidomics by LC-MS. *F*, sum of total ceramides in whole liver homogenates. (N = 3–4/group). *G*, sum of total ceramides in enriched liver mitochondria (N = 3/group). *H* and *I*, analysis of N-acyl chain d17 ceramides and d18 ceramides in mitochondria. The data presented are means ± SEM. Statistical analysis was performed using multiple unpaired *t* test. ∗*p* < 0.05. ER, endoplasmic reticulum; SPT3-hKO, SPTLC3 knockout.

To determine the contribution of Sptlc3 to the hepatocyte SL pool, targeted LC-MS/MS was performed to quantify specific SL species in livers from SPT3-hKO mice and controls. Because Sptlc3 generates sphingoid bases with atypical carbon numbers, we targeted SLs with sphingoid bases of 16, 17, 19, and 20 carbons, which are largely products of Sptlc1/3 complex, as well as the canonical 18-carbon bases (derived from both complexes) (33). Sphingoid bases serve as substrates for Ceramide synthases, which produce ceramides. Therefore, we measured ceramides comprised of an atypical/Sptlc3-derived base and the typical ceramide N-acyl chains. In whole liver homogenates, Sptlc3 depletion did not decrease ceramides with atypical bases. Rather, an increase was observed in standard ceramides with d18 and d19 backbones in SPT3-hKO livers (Fig. 4*F*). However, concentration of low-abundant SLs within distinct cellular compartments is an emerging theme in SL research. Recent studies demonstrate that in fact this compartmentalization of SLs can obscure changes in subcellular compartment pools when whole cell homogenates are used 34, 35. Therefore, SLs were measured in enriched mitochondria. In contrast to data from whole liver homogenates, in enriched mitochondria, Sptlc3 depletion reduced atypical ceramides by ∼50% relative to controls (Fig. 4*G*). In addition, a 10 to 15% decrease was observed in the ceramides with canonical (*i.e.*, d18) sphingoid bases, which is not unexpected, as both Sptlc3 and Sptlc2 can generate d18 sphingoid bases (Fig. 4*G*).

Among the atypical species, d17-ceramides were the most abundant in mitochondria. To determine whether specific ceramide synthases preferentially incorporated d17 bases, we analyzed the N-acyl chain distribution of d17 ceramides. Significant reductions were found in most d17-base ceramides (Fig. 4*H*). Similar reductions were seen in d16 ceramides; especially very long chain species; while d19 and d20 ceramides remained unchanged (Table S2). Canonical d18 ceramides also showed modest but consistent reductions in SPT3hKO mitochondria (Fig. 4*I*), these decreases were of less magnitude. This is not unexpected as Sptc1/3 also synthesizes d18-based SLs 33, 36. Together, these data demonstrate that Sptlc3-derived ceramides concentrate in mitochondria, where they are specifically required to support ATP production through complex I-III electron transport.

## Discussion

The liver holds a central position as a crucial metabolic hub orchestrating numerous physiological processes. Its multifaceted roles encompass the regulation of carbohydrate, lipid, as well as protein metabolism. In this intricate web of metabolic activities, SLs emerge as significant contributors to liver biology. Moreover, increasing evidence shows that SLs play crucial roles in mitochondrial biology, highlighting their influence on the structural integrity, physiology, and function. Within the context of mitochondria, SLs exhibit regulatory roles in processes such as mitochondrial fission and fusion, apoptosis signaling, and the modulation of mitochondrial membrane properties (17). Our study is the first to establish a critical role for Sptlc3 in supporting mitochondrial respiration, morphology, and functional integrity specifically in hepatocytes. While our lab previously demonstrated that cardiomyocyte-specific deletion of SPTLC3 results in intrinsic complex I enzymatic defect (29), the current work reveals a distinct mechanism in the liver: complex I retains intrinsic activity, but electron transfer is functionally impaired, highlighting an organ-specific divergence in Sptlc3’s role in mitochondrial function. Most notably, we uncover for the first time that Sptlc3 is essential for maintaining mitochondrial ceramide composition, which is required for optimal ETC function. These findings not only define a novel lipid-dependent mechanism regulating hepatocyte mitochondrial bioenergetics but also underscore the compartment- and tissue-specific functions of Sptlc3 and its noncanonical SL products.

Complex I, also known as NADH-ubiquinone oxidoreductase, is the largest component of the mitochondrial ETC and contributes nearly half of the proton motive force necessary for the synthesis of ATP in mitochondria 37, 38. Complex I is traditionally recognized as the entry point for electron transfer in the ETC, serving as a rate limiting step in respiration. It is necessary for basal respiration and maintenance of the NAD+/NADH ratio, which reflects cellular redox status (39). Interestingly, we have not observed any noticeable phenotypic changes or liver damage under basal conditions in SPT3-hKO mice, suggesting that despite the reduction in complex I activity, these mice can maintain normal liver function. Previous studies support this notion, as adult mice with conditional hepatocyte specific KO of *Ndufa9* (a core subunit of complex I) did not exhibit any abnormalities (40). However, this metabolic flexibility is not universal; complex I deficiencies in other tissues, particularly in the brain, can lead to severe mitochondrial disorders like Leigh’s syndrome, underscoring tissue-specific vulnerabilities (41). Hence, we propose that under basal conditions, these mice function adequately; however, when faced with high energy demands, or cellular stress such as in the case of HCC, these mice may exhibit protection from deleterious outcomes. This is supported by evidence demonstrating that inhibition of complex I in the ETC by compounds like rotenone and metformin inhibit the growth of HCC cell lines (42). Moreover, we show that SPTLC3-deficient liver cells ineffectively compensate for compromised mitochondrial function, by increasing glycolytic rates, enabling energy production through alternate pathways.

The observed decline in complex I activity within SPTLC3-deficient liver cells is not directly linked to a decrease in enzymatic function of complex I or impaired formation of supercomplexes in the respiratory chain. While further work is required to determine the precise molecular mechanisms by which loss of sptlc3-derived lipids compromise the function of ETC, our data suggest two potential mechanisms. The first being, mitochondria from SPT3-hKO mice display lower levels of CoQ9, the primary ubiquinone in mouse livers (32), this hints at the possibility that the observed results may arise in part from the limited availability of CoQ9. This observation is consistent with prior studies in *C. elegans* showing that complex I and complex II differ in their dependence on the endogenous ubiquinone pool and that loss of CoQ9 decrease complex I-III activity but not complex II-III activity (43). The second mechanism supports an organelle-specific role for enriched noncanonical SLs required for normal mitochondrial oxidation. In SPTLC3-deficient livers, the loss of mitochondrial d17 ceramides likely alters the mitochondrial membrane environment therefore hindering electron flow from complex I to complex III. However, further studies will be required to understand the mechanism by which d17 ceramides regulate levels of CoQ9.

Glycerophospholipids, and cardiolipin in particular, are well established as core structural and functional components of the mitochondrial inner membrane; they support cristae architecture, stabilize respiratory chain supercomplexes, and interact directly with ETC proteins, including complex I, III, and IV 44, 45, 46, 47, 48. While these canonical roles are well characterized, our findings define a distinct and previously unrecognized function for Sptlc3-derived atypical SLs in mitochondrial bioenergetics. Whether these atypical species act directly on respiratory complexes, in a manner analogous to cardiolipin, or instead act indirectly by supporting the overall lipid composition and biophysical properties of the mitochondrial membrane, remains to be determined; these mechanisms are not mutually exclusive and could act in concert. It is also possible that this relationship is bidirectional: the ultrastructural mitochondrial abnormalities observed in SPT3-hKO mice, including reduced cristae density, could themselves impair membrane lipid maintenance and turnover, since a loss of cristae membrane would be expected to reduce the overall lipid content of the organelle independent of any direct SL-mediated effect. Distinguishing among these possibilities will require future studies directly testing lipid-protein interactions, such as native mass spectrometry or reconstitution assays with purified ETC complexes.

Overall, our study on SPTLC3 in the liver reveal in hepatocytes, Sptlc3-dependent SLs are essential for maintaining the atypical ceramide composition of the mitochondrial membrane, which supports efficient electron transfer from complex I, and with its loss leading to reduced ATP production and a concomitant metabolic shift toward glycolysis. This contrasts with a previous study from our lab in cardiomyocytes, where sptlc3 loss resulted in an intrinsic complex I defect rather than altered electron flow, despite a similar decrease in mitochondrial oxidation and compensatory increase in glycolysis. The contrast in mechanism of Complex I compromise between cardiomyocytes and hepatocytes indicates cell-type specific mechanisms for impact on cell metabolism. Moreover, in the previous study we identified lactosylceramides associated with cardiomyocyte complex I, which we did not find in this study, suggesting distinct membrane lipid composition in mitochondria from different organs. These tissue specific differences highlight the importance of organ- and organelle-specific considerations when targeting bioactive SLs in metabolic regulation and disease. Given that hepatocellular carcinoma cells frequently reprogram their lipid and mitochondrial metabolism to support proliferation, an intriguing direction for future work is the role of SPTLC3 in liver cancer. We found that SPTLC3 expression is elevated in the hepatocellular carcinoma cell lines HepG2 and Huh7 relative to the non-transformed hepatocyte line HC3716, and that SPTLC3 knockdown selectively reduced viability in the cancer cell lines without affecting HC3716 cells (Fig. S3, *A* and *B*), suggesting that hepatocellular carcinoma cells may be selectively dependent on SPTLC3, potentially to support their elevated energy demands; a detailed mechanistic and mitochondrial characterization of SPTLC3 in this cancer context is the focus of a separate article currently in preparation. Taken together, our findings identify SPTLC3-derived atypical SLs as essential, nonredundant regulators of hepatocyte mitochondrial ceramide composition and ETC function, distinct in both origin and role from the canonical SL pool generated by SPTLC1/SPTLC2. Given the central position of the liver in systemic lipid and energy homeostasis, these findings establish SPTLC3 as a previously unrecognized node linking SL metabolism to mitochondrial bioenergetics, with relevance extending from hepatic metabolic disease to hepatocellular carcinoma.

## Experimental procedures

### Generation of SPT3-hKO mice

All animal experiments were conducted under the guidelines of the “Guide for the care and use of laboratory animals” published by National Institutes of Health (revised 2011). The study protocol was approved by the Virginia Commonwealth University Institutional Animal Care and Use Committee (AD10001921). The SPTLC3 floxed mouse was created by Dr David Montefusco as described earlier (28).

### Isolation of primary mouse hepatocytes

Isolation was carried out based on the method of Seglen *et al.* (45). Briefly, the portal vein was catheterized under isoflurane anesthesia and the liver was flushed with heparin, EGTA perfusate, and then Collagenase perfusate. Hepatocytes were isolated by Percoll gradient and cultured in supplemented William’s E media.

### Western blotting

Primary murine hepatocytes and HC-3176 cells were harvested into 200 μl per well of RIPA buffer containing Pierce protease and phosphatase inhibitors. Samples were quantified by Pierce BCA assay and prepared in 4 x Laemelli buffer at 2 mg/ml, and 15 μg was loaded per well of a 26-lane 4 to 15% Biorad criterion gradient gel. Protein was transferred for 10 min onto a PVDF membrane using Biorad Trans-Blot Turbo transfer system. The membranes were blocked and blotted in 5% bovine serum albumin (BSA) in Tris-buffered saline with Tween-20 for one hour followed by overnight incubation in primary antibodies at 4 °C. Antibodies used were - SPTLC3 (Thermo Fisher Scientific, PA5-65493), SPTLC2 (Thermo Fisher Scientific, PA5-21142), Vinculin (Cell Signaling Technology, 13901), COXVI (Cell Signaling Technology, 4850), and Calreticulin (Cell Signaling Technology, 2891) for mouse tissue and cells. For human cell lines SPTLC3 (Cloud-Clone Corp., PAH136Hu01) was used at 1:1000 dilution in Tris-buffered saline with Tween-20. The secondary antibody used was anti-rabbit HRP (Cell Signaling Technology, 7074), blotted in 1:5000 dilution at RT for 1 h.

### Quantitative real-time PCR

Total RNA was extracted from primary hepatocytes using TRIzol reagent (Invitrogen, 15596026) followed by purification with the RNeasy Mini Kit (Qiagen, 74106) according to the manufacturers’ instructions. RNA concentration was determined using a NanoDrop 2000 spectrophotometer. cDNA was synthesized from 1 μg total RNA using the iScript Advanced cDNA Synthesis Kit (Bio-Rad, 1708890). Quantitative real-time PCR was performed on a CFX96 Real-Time PCR System (Bio-Rad) using SSoAdvanced SYBR Green Supermix (Bio-Rad, 1725272). Gene expression was normalized to the geometric mean of the reference genes Hprt and 18S rRNA and calculated using the ΔΔCt method. Primer sequences were as follows.

**Table:** 

Name	Forward primer 5′ - 3′	Reverse primer 5′ - 3′
SPTLC1	CTATGCAGATACTCTGTGTGTGT	GTATGAAGATCTGTTAGTGAAGCTC
SPTLC2	GAGTCCAGAGCCAGGTTTTG	CTGAGGGAGCACCAAAAAG
Actb	GGCTGTATTCCCCTCCATCG	CCAGTTGGTAACAATGCCATGT

### Proteomics mass spectrometry

The proteomic sample preparation was done using commercially available Pre-omics iST kit (P.O.00027). LC-MS/MS analysis were performed using a 480 Exploris tandem mass spectrometer (Thermo Fisher Scientific) coupled to a Neo Vanquish nanoflow UPLC system (Thermo Fisher Scientific). The LC-MS/MS system was fitted with an Easy spray ion source and an Acclaim PepMap 300 μm × 5 mm nanoviper C18 5 μm × 100 Å precolumn in series with an Aurora XT 75um × 250 mm C18 1.7um (Ion Optick). The mobile phase consists of Buffer A (0.1% formic acid in water) and Buffer B (80% acetonitrile in water, 0.1% formic acid). 2μl of peptides were injected onto the above column assembly and eluted with an acetonitrile/0.1% formic acid gradient at a flow rate of 350 nl/min over 2 h. The nano-spray ion source was operated at 1.9 kV. The digests were analyzed with a data independent acquisition method acquiring a full scan mass spectrum (350–1200 m/z), at an AGC = 300% and an ITmax = Auto followed by targeted MS2 analysis with 23.4 Da window. NCE = 30%, AGC = Custom 1000%, and ITmax = Auto.

The data were analyzed in Spectronaut (ver. 21.00) using the Pulsar search algorithm and the Human and Contaminant database. Proteins were identified at an FDR <0.01 and quantification used the MS2 level peptide intensities.

### RNA sequencing

Liver tissues from SPT3-hKO and Alb-cre mice were homogenized using magnetic bead homogenizer in 1 ml of Trizol (Invitrogen, 15596026) followed by RNeasy mini kit (Qiagen, 74106) extraction and column purification. RNA concentration was measured using Nanodrop 2000. A total of 1 μg of RNA was used for RNA sequencing. Only RNA with an RNA integrity value ≥ 8.0 was used for library preparation. The RNA-Seq library with poly A tail enrichment was prepared using the Illumina NovaSeq sample preparation kit and subjected to two rounds of sequencing on the Illumina NovaSeq 6000 PE150 platform. RNA-Seq libraries were pooled together to aim for 25-40M read passed filtered reads per sample. All sequencing reads were quality controlled using FastQC v0.11.2. Illumina adapters were trimmed using trimmomatic v.0.33, replicates were merged and aligned with UCSC mouse genome build mm10 using STAR v. 2.5.2b. The BAM files from alignment were processed using featureCounts v1.4.6-p4 with GRCm38 v.100 gene annotations for mouse cells to obtain the counts per gene in all samples. The differential expression analysis was performed using edgeR v3.18.1. Genes having counts per million less than 2 in all samples were excluded. Differentially expressed genes were defined using *p* value < 0.01 and |log2fold change| > 0.5 cutoff thresholds. All bioinformatics analyses such as GO were conducted in R/Bioconductor computing environment v3.4.0. GO overrepresentation analysis (Fisher's exact test) was performed on the list of differentially expressed genes against a background of all detected genes using Novogene's in-house bioinformatics pipeline The underlying differential expression dataset has been deposited in NCBI GEO, accession number GSE339084.

### Measurement of oxygen consumption using seahorse

Primary hepatocytes from Alb-cre and SPT3-hKO mice and Hc3716 cells were harvested and plated on collagen coated 24 well seahorse plates. Mitochondrial respiration and glycolysis were measured using Agilent Seahorse XFe24 Analyzer following Agilent’s mito-stress test and glycolytic rate test protocols respectively. Concentrations for Mito-stress test are as follows: Oligomycin 1.5 μM, FCCP 1 μM, and Rotenone/Antimycin A 2.0 μM. For glycolytic rate test 10 mM of glucose, 1.0 μM of oligomycin and 50 mM of 2-DG were used.

ETC complex activity (I, II, and IV) were assessed using previously described protocol (30); 100 μg of frozen-thawed mitochondria from liver tissue was loaded on to a Seahorse XF24 plate in 100 μl of 1x MAS buffer (1X MAS buffer (Mannitol 220 mM, sucrose 70 mM, KH_2_P0_4_ 5 mM, Hepes 2 mM, EGTA 1 mM, fatty free BSA 0.2% (W/V)). The loaded plate was centrifuged at 2000 *g* for 5 min at 4 °C (no brake) followed by additional 400 μl of MAS to each well. The Seahorse injections were programed as follows: Port A NADH (1 mM), or Succinate (5 mM) + Rotenone (2 μM); Port B Rotenone (2 μM) + Antimycin A (4 μM); Port C TMPD (0.5 mM) + Ascorbic acid (1 mM); Port D Azide (50 mM). The calculations for each complex were as follows Complex I maximal respiration OCR_NADH_ – OCR_ROT + AA_, complex II maximal respiration OCR_succ + ROT_ – OCR_ROT/AA_, complex IV OCR_TMPD + ASC_- OCR_Azide_. All the assays performed on cell lines were normalized by cell count after 4′,6-diamidino-2-phenylindole (DAPI) staining and for assays done on mitochondria, 100 μg of sample was loaded on to each well.

### Light and TEM

Protocol from our collaborator Dr Sushil Mahata was used to fix liver sections for TEM (49). Mice were deeply anesthetized and perfused through the left ventricle. Mice were flushed for approximately 2 min with a prewarmed (37 °C) calcium and magnesium free buffer, composed of DPBS (Life Technologies), 10 mM Hepes, 0.2 mM EGTA, 0.2% BSA, 5 mM glucose and 10 mM KCl. Freshly prepared prewarmed (37 °C) fixative containing 2.5% glutaraldehyde, 2% paraformaldehyde in 0.15 M cacodylate buffer was used to perfuse mice. Liver sections were dissected out, stored at RT or 4 °C. The samples were postfixed in 1% OsO4 in 0.1 M cacodylate buffer for 1 h on ice. Afterward, they were stained with 2 to 3% uranyl acetate for 1 h on ice. The cells were then dehydrated using a series of ethanol washes (20–100%) on ice, followed by one wash with 100% ethanol and two washes with acetone, each lasting 15 min. Subsequently, the samples were embedded with Durcupan (Sigma-Aldrich, 44611). Ultrathin sections of 50 to 60 nm thickness were cut from the embedded samples using a Leica UCT ultramicrotome and collected on Formvar and carbon-coated copper grids. These sections were stained with 2% uranyl acetate for 5 min and Sato's lead stain for 1 min. The grids were observed using a JEOL JEM1400-plus TEM equipped with a Gatan OneView digital camera having a resolution of 4 k × 4 k (Gatan). To analyze the images, the mitochondrial area was determined using the free-hand tool in ImageJ by manually tracing around the outer membrane of the mitochondria. In addition, the area of each crista membrane was calculated using the same method. The sum of the areas of all the cristae was then divided by the sum of the mitochondrial area, resulting in the calculation of the cristae volume density. Each section had one to two mitochondria, TEM was used to assess mitochondrial morphology. Each section contained 1 to 2 mitochondria, and 25 to 46 sections were analyzed per group. For the control group, three biological replicates (n = 3) were used. For the SPT3-hKO group, two biological replicates (n = 2) were included, as the third sample lacked distinguishable cristae, precluding quantitative assessment.

### mtDNA copy number

For DNA isolation from primary hepatocytes and HC-3716 cell line Qiagen, DNeasy Blood & Tissue kit, 69504 was used. For each sample, 5 ng of DNA was added to each well of a qPCR plate. The Cq values of the mitochondrial gene MT-CO2 (Fwd: CCATAGGGCACCAATGATACTG, Rev AGTCGGCCTGGGATGGCATC) were determined, and the Cq values of the nuclear gene 18s RNA (Fwd: CTTAGAGGGACAAGTGGCGTTC, Rev CGCTGAGCCAGTCAGTGTAG) were also measured. To determine the mtDNA copy number the following formulas were used: (a) ΔC_T_ = (nucDNA C_T_ − mtDNA C_T_). (b) Relative mtDNA content = 2 × 2^ΔCT^.

### Flow cytometry

HC3716 cells and enriched mitochondria were used for flow cytometry. After gating for single cells, hepatocytes were analyzed for MitoTracker Green (Cat# M7514), TMRE (Cat# ab113852). Multicolor data acquisition was performed using Cytek Aurora (Cytek). Data are analyzed using FlowJo, version 10.8.0.

### MitoTracker Green and TMRE staining

Cells were detached from the plate by incubating in 2 mM EDTA in PBS and trypsin for 20 min. After the cells were completely detached, they were centrifuged at 10,000*g* for 5 min. The pellet was resuspended in 100 nM MitoTracker Green (M7514) and 10 nM TMRE (ab113852) in Krebs Ringer Buffer (KRB – 125 mM NACl, 5 mM KCl, 1.3 mM CaCl, 1.2 mM MgSO_4_, 25 mM Hepes pH 7.4, and 2% BSA). Flow cytometry was performed on the samples after 30 min incubation at 37 C.

### Oroboros

Murine primary hepatocytes; approximately 1 to 2 × 10^6^ cells; were suspended in MiR05 mitochondrial respiration medium. The medium consisted of 0.5 mM EGTA, 110 mM sucrose, 3 mM MgCl_2_, 20 mM taurine, 10 mM KH_2_PO_4_, 20 mM Hepes (pH 7.1), and 1 g/L BSA. Were cells digitonin permeabilized?? To determine oxidative phosphorylation, a multiple substrate-uncoupler-inhibitor titration protocol was optimized using a high-resolution oxygraph (Oxygraph-2k; Oroboros, Innsbruck, Austria). Glutamate/Pyruvate/malate (10 mM, 5 mM, and 2 mM, respectively) were utilized as complex I substrates. After permeabilization of hepatocytes with digitonin (10 μg/ml), oxygen consumption was strongly activated by 2 mM ADP. Sequential additions of rotenone (0.5 μM), succinate (a complex II substrate; 10 mM), TTFA (40 μM), TMPD/ascorbate (complex IV substrates; 0.3 mM and 3 mM, respectively), and azide (15 mM) were made to determine the sensitive rates of oxidative phosphorylation using complex I, II, and IV substrates, respectively. Protocol adapted from 50, 51. The evaluation of oxygen consumption was performed using the Oroboros DatLab4 software, and the results were expressed as nanomoles of oxygen per minute per milligram.

### ATP detection assay

Primary hepatocytes from Alb-cre and SPT3hKO male mice were seeded on a 96 well collagen coated plate. Luminescent ATP detection kit from Abcam (ab113849) was used to measure ATP levels within the cells. The assay involves lysis of the cells and addition of luciferase enzyme and luciferin. After 10 min of dark incubation, sample reactions were transferred to a 96 well opaque plate and read using a microplate luminometer. Cells in each well were counted using Lionheart FX after DAPI staining and luminescence from each well was normalized with cell count.

### NADH/NAD Glo assay

A bioluminescent kit from Promega (G9071) was used for detecting total oxidized and reduced nicotinamide adenine dinucleotides (NAD+ and NADH respectively). Like ATP detection assay, at the last step sample reactions were transferred to a 96 well opaque plate and read with a microplate luminometer. Cells in each well were counted using BioTek Lionheart FX after DAPI staining and luminescence from each well was normalized with cell count.

### Blue native polyacrylamide gel electrophoresis

Enriched liver mitochondria were solubilized in 1% Triton X-100 before separation of complexes by BN-PAGE. Proteins were either stained with 0.5% Coomassie Blue R (Sigma-Aldrich) in 40% ethanol, 7% acetic acid or transferred to PVDF membrane (Thermo Fisher Scientific, BN1002BOX) for immunodecoration. Membranes were probed with antibodies against the 70-kDa subunit of CII (Invitrogen) or against the CI subunit NDUFA9.

### ETC assays

To measure the activities of Complex I, Complex II, Complex I-III, Complex II-III, and Complex IV, mitochondrial fractions from both control and SPT3-hKO samples were utilized. The protocols outlined in Kirby *et al.* (2007) were followed with certain modifications (52). For Complex I activity assessment, no BSA was included in the media, and for Complex I-III evaluation, sodium succinate and rotenone were substituted with 0.3 mM NADH.

### LC-MS/MS analysis of CoQ9 and CoQ10

The LC-MS/MS analysis of CoQ9 and CoQ10 was performed on an AB SCIEX QTRAP 6500 system including a SHIMADZU (Kyoto, Japan) Nexera ultra high-performance liquid chromatography system and a hybrid triple quadrupole and linear ion trap mass spectrometer. The standard stock solution of CoQ9 and CoQ10 was prepared in methanol with the final concentration of 10 mM and stored at −80 °C. Calibration standards were prepared by serial dilution of the stock solutions with methanol within the range from 0.005 μM/L to 10 μM/L. Standard mixture of CoQ9 and CoQ10 with final concentration of 0.1 μM/L was used as quality control (QC) sample to inject before and after the calibrations and between each group of samples to monitor the drift of intensity. Chromatographic separation of CoQ9 and CoQ10 was achieved on a Synergi Polar-RP Synergi Polar-RP 20 x 150 mm (4 μm, 80A) column (Phenomenex, Torrance, CA) with isocratic elution for 5 min at the flow rate of 0.6 ml/min with the oven temperature of 30 °C. A final concentration of 5 mM ammonium formate in methanol was used for mobile phase 53, 54. The injection was 10 μl for all samples and calibration standards. The transition of 812.4 > 197.4 was used for CoQ9 and 880.4 > 197.4 for CoQ10. The collision energy, declustering potential and cell exit potential were manually tuned and optimized with QC sample. Analyst 1.6 software was used for system control and data acquisition; MultiQuant 3.0 software was used for data processing and quantitation. Equal amount of mitochondrial protein was used as starting material.

### HC3716-hTERT cell line and siRNA treatment

Immortalized human fetal hepatocyte cell lines (HC3716-hTERT) were acquired from our collaborator Dr Devanand Sarkar’s laboratory. Cells were seeded on collagen coated dishes with hepatocyte culture medium (Lonza CC-3198; HBM + HCM supplement kit) + 2% DMSO + 5% FBS + 10% human serum and 1% Pen-strep. For propagating cells, cells were washed twice and incubated for 10 min with 0.025% trypsin and then split 1:10 ratio. For siRNA treatments, when the cell reached 50 to 70% confluency, they were subjected to specific siRNA treatments. For the negative control, Allstars negative control siRNA (Qiagen, SI03650318) was utilized. In addition, for the Sptlc2 (Hs_SPTLC2_1, Qiagen SI00732795) and Sptlc3 treatments (Hs_SPTLC3_2, Qiagen SI04026421 and Hs_SPTLC3_5, Qiagen SI04179770), Flexitube siRNA Premix (Qiagen, 1027418) were used. For FRAP experiments, Alexa Fluor-tagged siRNAs were utilized, including Allstars negative control 488 siRNA Alexa Fluor (Qiagen, SI03650318|S2|504), Hs_SPTLC2_1647 Alexa Fluor (Human SPTLC2 siRNA, Qiagen, SI00732795|S2|507), and Hs_SPTLC3_2488 Alexa Fluor (Human SPTLC3 siRNA #1, Qiagen, SI04026421|S2|504). The transfection process was carried out using Lipofectamine RNAiMAX transfection reagent (Thermo Fisher Scientific, 13778150) following the instructions provided by the manufacturer.

### FRAP

Live cells (HC3716 transfected with pDsRed2-Mito vector (Clontech)) were imaged with an inverted Zeiss LSM880 confocal microscope equipped with 63x PlanApo oil immersion lens (NA 1.4) and stage microincubator (37C, 5% CO2). Fluorescence was excited with a 561 nm DPSS laser (25 mW). Emission was detected in the 570 to 625 nm range. Confocal pinhole was set to 1 Airy unit for 600 nm emission wavelength. Pixel size was set to 144 nm in (xy). The images (96 × 96 pixels) were registered with 1.3 μs pixel dwell time (bidirectional scanning) and 16 bit precision. Time series of images were collocated with 16 ms frame time. Laser power was adjusted so that the dynamic range was filled in 50% with the detector (PMT) gain of 700 V. Prior to image collection the frame was centered on a single (isolated) mitochondrion. FRAP protocol was implemented with 10 prebleach, 1 bleach, and 300 postbleach frames. Image collection was executed at 0.1% of laser power. Full power and spot illumination was used for bleaching. Fluorescence was integrated over an illumination PSF area (5 pixel diameter circle) and following background subtraction and double normalization as described in (55) single exponential was fitted to the fluorescence recovery curve (rate represented with the half-time).

### Mitochondrial isolation

The livers from Alb-Cre and SPT3-hKO mice were subjected to a series of steps for mitochondrial isolation. Firstly, the livers were washed twice with MSM-E buffer, pH 7.4, composed of 220 mM mannitol, 70 mM sucrose, 5 mM MOPS, and 2 mM EDTA. After washing, they were gently dried using blotting paper, weighed, and subsequently homogenized using 5 ml of MSM-E buffer per gram of liver. The homogenization process involved two strokes with a loose-fitting pestle. The resulting homogenate was then centrifuged at 350*g* for 10 min, and the supernatant was carefully collected. This supernatant was subjected to a second round of centrifugation at a higher speed of 7000*g* for 10 min. The pellet was resuspended in 5 ml of MSM-E buffer per gram of liver and subjected to another round of centrifugation at 7000*g* for 10 min. The resulting pellet was saved once again. Following this, the pellet obtained from the previous step was resuspended in approximately 0.2 ml of MSM-E buffer per gram of liver. The protein concentration in the final suspension was determined using either the BCA or Lowry protocol.

### LC-MS/MS-targeted lipidomics

Liver homogenates and enriched mitochondria were subjected to LC-MS/MS targeted lipidomics. BCA was performed so that starting material for each sample was 4 mgs of protein (N = 3–4/group). Samples were sent for targeted lipidomics after Bligh Dyer extraction. Each sample had 10 μl of the VCU metabolomics core internal standards (homemade recipe comprised of Avanti Polar Lipid standards d17:1 So sphingosine, d17:0 Sa dihydrosphingosine, d17:1 So1P sphingosine-1-phosphate, d17:0 Sa1P dihydrosphingosine-1-phosphate, C12 ceramide, C12 monohexosylceramide, C12 sphingomyelin, and C12 lactosylceramide). All samples were run on AB Sciex 5500 QTrap (GenTech Scientific).

### LC-MS/MS-untargeted lipidomics

Extracted samples were reconstituted in 150 μl methanol, followed by addition of 10 μl SPLASH Lipidomix internal standard to each sample. A pooled QC was prepared by combining equal volumes of all samples and injected periodically to condition the column and monitor instrument stability. Samples (10 μl) were analyzed using a Vanquish UHPLC coupled to a Q-Exactive HF mass spectrometer (Thermo Fisher Scientific) with an Accucore Vanquish C18 column (150 × 2.1 mm, 1.5 μm) at 55 °C. Mobile phases consisted of (A) acetonitrile:water (50:50) and (B) isopropanol:acetonitrile:water (88:10:2) with 5 mM ammonium formate and 0.1% formic acid. The flow rate was 0.26 ml/min with a gradient from 1% to 100% B over 20 min, followed by reequilibration. Data were acquired using HESI-II in positive and negative modes (scan range 300–2000 m/z, resolution 240,000) with ddMS2 (Top10) at 30,000 resolutions. For data processing, LipidSearch (v4.2.21) was used for alignment and annotation with QC-based filtering (RSD <30%, ≥80% presence). Intensities were QC-corrected and normalized using the MSTUS approach. Statistical analysis included PCA in MetaboAnalyst 5.0, Tukey *post hoc* testing with FDR correction, and hierarchical cluster analysis and heatmap were generated with the “euclidean” algorithm and “ward D2” method under the “pheatmap” package with R program (version 4.2).

### Cell viability assay (CCK8)

Cell viability was assessed using the Cell Counting Kit-8 (CCK-8; Sigma-Aldrich, 96992) according to the manufacturer's instructions. Cells were seeded in 96-well plates and transfected with control (si-Cntrl) or SPTLC3-targeting siRNA; 24 h posttransfection, CCK-8 reagent was added to each well and incubated for 1 to 2 h at 37 °C, and absorbance was measured at 450 nm using a microplate reader. Viability was normalized to the si-Cntrl condition, set to 100% for each cell line.

### Statistical analysis

Statistical analyses were performed using GraphPad Prism 10 version 10.1.2. All values are reported as mean ± SEM. When comparing two groups, pairwise comparisons of normally distributed datasets were conducted using a Welch's corrected two-tailed Student *t* test. For comparisons involving multiple groups, either a one-way ANOVA or a two-way ANOVA with the appropriate correction test was performed.

## Data availability

The RNA-seq data generated in this study have been deposited in the NCBI Gene Expression Omnibus (GEO) database under accession number GSE339084 and will be made publicly available upon publication of this article.

## Supporting information

This article contains supporting information.

## Conflict of interest

The authors declare that they have no conflicts of interest with the contents of this article.
