# Supplementary material for: Hepatocyte serine palmitoyltransferase 3 is essential for mitochondrial ceramide homeostasis and electron transport chain function
Source: J Biol Chem. 2026 Oct 9;302(12):113630. doi: 10.1016/j.jbc.2026.113630 (PMC13760969; doi:10.1016/j.jbc.2026.113630)
Supplement: Supplementary file 1 [file mmc1.docx]

**Supplemental Figure S1: Validation of Hepatocyte-Specific Knockout of SPTLC3 in Mice**

**a.** Schematic representation of the generation of the SPT3-hKO murine model using the cre-lox system. **b.** Genotyping results of tail snips confirming the successful deletion of the SPTLC3 gene. **c.** qPCR of SPTLC2 and SPTLC3 in primary hepatocytes from Alb-cre and SPT3hKO mice (N=6/group). The data presented are means ± SEM. Statistical analysis performed using unpaired Student's t-test. *P<0.05, **P<0.01, ***P<0.001, ****P<0.0001. **d.** SPTLC1 was immunoprecipitated from HC3716 hepatocyte lysates and immunoprecipitates were analyzed by proteomics mass spectrometry (N=4 independent replicates).

**Supplemental Figure S2: Validation of SPTLC3 Knockdown Using Two Independent siRNA Sequences in HC3716 Hepatocytes**

**a.** Western blot showing SPTLC3 protein expression in HC3716 cells treated with media alone, negative control siRNA (si-Cntrl), or one of two independent SPTLC3-targeting siRNAs (si-SPT3-2, si-SPT3-5); GAPDH was used as a loading control (N=3/treatment). **b.** Seahorse Mito Stress Test showing normalized oxygen consumption rate (OCR) over time in HC3716 cells treated with control siRNA (si-control-1) or either of the two independent SPTLC3 siRNAs (si-SPT3-2, si-SPT3-5), confirming that the reduction in OCR is reproduced with both siRNA sequences and is not attributable to an off-target effect of either sequence alone (N=5/treatment). The data presented are means ± SEM. Statistical analysis performed using one-way ANOVA with multiple comparisons. ****P<0.0001.

**Supplemental Figure S3: SPTLC3 Expression and Its Role in Hepatocellular Carcinoma Cell Viability**

**a.** Western blot showing SPTLC3 protein expression in the non-transformed human hepatocyte cell line HC3716 and the hepatocellular carcinoma cell lines HepG2 and Huh7; vinculin and total protein were used as loading controls (N=2/group). **b.** Cell viability (%) 24 hours after transfection with control siRNA (si-Cntrl) or SPTLC3-targeting siRNA (si-SPTLC3) in HepG2, Huh7, and HC3716 cells, with viability normalized to si-Cntrl, set to 100% for each cell line. SPTLC3 knockdown significantly reduced viability in HepG2 and Huh7 cells but had no effect on HC3716 cells. The data presented are means ± SEM. Statistical analysis performed using unpaired multiple t-tests (N=16/group). **P<0.01, ****P<0.0001, ns=not significant.

**Supplemental Table S1.** Untargeted lipidomic profiling of mitochondrial fractions isolated from Alb-Cre and SPT3-hKO liver, showing only lipid species that differed significantly between genotypes.

| **LipidMolec** | **Class** | **SPT3-hKO** | | | | **Alb-cre** | | | | **Ttest** |
| --- | --- | --- | --- | --- | --- | --- | --- | --- | --- | --- |
| **AEA(16:0)** | AEA | 6E+06 | 4E+06 | 7E+06 | 4E+06 | 8E+06 | 8E+06 | 9E+06 | 7E+06 | 0.013 |
| **Cer(t36:0)** | Cer | 5E+07 | 4E+07 | 4E+07 | 4E+07 | 8E+07 | 7E+07 | 7E+07 | 6E+07 | 4E-04 |
| **Cer(d18:1_23:0)** | Cer | 9E+06 | 8E+06 | 8E+06 | 7E+06 | 1E+07 | 1E+07 | 1E+07 | 1E+07 | 0.002 |
| **Cer(d18:1_24:0+O)** | Cer | 1E+07 | 8E+06 | 8E+06 | 8E+06 | 2E+07 | 1E+07 | 2E+07 | 1E+07 | 0.007 |
| **Cer(d18:2_23:0)** | Cer | 1E+07 | 1E+07 | 1E+07 | 1E+07 | 2E+07 | 2E+07 | 2E+07 | 2E+07 | 0.012 |
| **Cer(t18:0_24:0)** | Cer | 7E+06 | 6E+06 | 6E+06 | 6E+06 | 1E+07 | 1E+07 | 9E+06 | 7E+06 | 0.014 |
| **Cer(d18:2_24:2)** | Cer | 3E+07 | 3E+07 | 3E+07 | 3E+07 | 4E+07 | 5E+07 | 5E+07 | 3E+07 | 0.014 |
| **Cer(d18:1_25:1)** | Cer | 8E+08 | 6E+08 | 5E+08 | 5E+08 | 9E+08 | 8E+08 | 8E+08 | 7E+08 | 0.027 |
| **Cer(t41:0)** | Cer | 2E+08 | 1E+08 | 1E+08 | 1E+08 | 3E+08 | 2E+08 | 2E+08 | 2E+08 | 0.035 |
| **Cer(d18:0_24:0)** | Cer | 8E+06 | 6E+06 | 5E+06 | 4E+06 | 1E+07 | 9E+06 | 8E+06 | 7E+06 | 0.044 |
| **Cer(d18:1_26:0)** | Cer | 4E+07 | 3E+07 | 2E+07 | 2E+07 | 5E+07 | 4E+07 | 4E+07 | 3E+07 | 0.047 |
| **CL(18:2_18:2_18:2_22:6)** | CL | 1E+07 | 4E+06 | 7E+06 | 7E+06 | 2E+07 | 2E+07 | 2E+07 | 1E+07 | 0.003 |
| **CL(18:2_18:2_20:3_18:2)** | CL | 6E+06 | 4E+06 | 3E+06 | 3E+06 | 2E+07 | 2E+07 | 1E+07 | 5E+06 | 0.02 |
| **CL(18:2_18:2_18:2_20:4)** | CL | 3E+06 | 3E+06 | 2E+06 | 3E+06 | 3E+06 | 2E+06 | 2E+06 | 2E+06 | 0.038 |
| **CL(20:2_18:2_18:2_18:2)** | CL | 2E+06 | 1E+06 | 2E+06 | 2E+06 | 2E+06 | 2E+06 | 2E+06 | 3E+06 | 0.041 |
| **cPA(18:0)** | cPA | 1E+07 | 8E+06 | 1E+07 | 1E+07 | 2E+07 | 2E+07 | 2E+07 | 1E+07 | 0.014 |
| **DG(18:1_24:1)** | DG | 2E+07 | 2E+07 | 2E+07 | 1E+07 | 6E+07 | 5E+07 | 6E+07 | 4E+07 | 1E-04 |
| **DG(20:0_18:1)** | DG | 6E+06 | 6E+06 | 3E+06 | 4E+06 | 2E+07 | 1E+07 | 2E+07 | 2E+07 | 1E-04 |
| **DG(18:0_17:0)** | DG | 2E+07 | 1E+07 | 1E+07 | 1E+07 | 3E+07 | 3E+07 | 4E+07 | 3E+07 | 2E-04 |
| **DG(18:3_18:2)** | DG | 3E+07 | 3E+07 | 3E+07 | 2E+07 | 8E+07 | 7E+07 | 8E+07 | 6E+07 | 3E-04 |
| **DG(20:5_18:2)** | DG | 4E+07 | 3E+07 | 4E+07 | 3E+07 | 9E+07 | 9E+07 | 1E+08 | 8E+07 | 4E-04 |
| **DG(18:1_22:0)** | DG | 1E+07 | 1E+07 | 1E+07 | 1E+07 | 2E+07 | 2E+07 | 2E+07 | 2E+07 | 4E-04 |
| **DG(20:1_18:1)** | DG | 3E+07 | 2E+07 | 1E+07 | 1E+07 | 5E+07 | 5E+07 | 6E+07 | 4E+07 | 5E-04 |
| **DG(22:0_18:2)** | DG | 2E+07 | 2E+07 | 1E+07 | 1E+07 | 4E+07 | 3E+07 | 5E+07 | 4E+07 | 6E-04 |
| **DG(20:2_18:2)** | DG | 4E+08 | 3E+08 | 2E+08 | 2E+08 | 7E+08 | 7E+08 | 8E+08 | 5E+08 | 0.001 |
| **DG(18:1_20:4)** | DG | 1E+09 | 8E+08 | 7E+08 | 7E+08 | 2E+09 | 2E+09 | 2E+09 | 1E+09 | 0.002 |
| **DG(18:1_24:0)** | DG | 4E+06 | 1E+06 | 2E+06 | 2E+06 | 8E+06 | 9E+06 | 1E+07 | 6E+06 | 0.002 |
| **DG(18:0_16:0)** | DG | 8E+07 | 7E+07 | 7E+07 | 7E+07 | 1E+08 | 1E+08 | 1E+08 | 1E+08 | 0.003 |
| **DG(16:1_20:4)** | DG | 3E+07 | 3E+07 | 2E+07 | 2E+07 | 5E+07 | 4E+07 | 5E+07 | 4E+07 | 0.003 |
| **DG(16:0_18:3)** | DG | 1E+07 | 1E+07 | 1E+07 | 9E+06 | 2E+07 | 2E+07 | 2E+07 | 2E+07 | 0.004 |
| **DG(16:0_18:2)** | DG | 1E+07 | 1E+07 | 1E+07 | 1E+07 | 2E+07 | 2E+07 | 2E+07 | 2E+07 | 0.004 |
| **DG(15:0_18:2)** | DG | 8E+07 | 8E+07 | 6E+07 | 7E+07 | 1E+08 | 1E+08 | 1E+08 | 9E+07 | 0.004 |
| **DG(18:1_18:1)** | DG | 2E+07 | 1E+07 | 8E+06 | 8E+06 | 2E+07 | 3E+07 | 3E+07 | 2E+07 | 0.005 |
| **DG(18:0_18:0)** | DG | 1E+07 | 1E+07 | 6E+06 | 5E+06 | 2E+07 | 2E+07 | 2E+07 | 2E+07 | 0.005 |
| **DG(18:1_22:5)** | DG | 6E+07 | 5E+07 | 5E+07 | 4E+07 | 9E+07 | 8E+07 | 1E+08 | 7E+07 | 0.006 |
| **DG(16:0_20:4)** | DG | 5E+08 | 6E+08 | 4E+08 | 3E+08 | 9E+08 | 7E+08 | 8E+08 | 6E+08 | 0.008 |
| **DG(18:0_20:3)** | DG | 4E+06 | 3E+06 | 2E+06 | 2E+06 | 7E+06 | 6E+06 | 9E+06 | 5E+06 | 0.009 |
| **DG(16:0_22:5)** | DG | 7E+06 | 5E+06 | 3E+06 | 4E+06 | 1E+07 | 7E+06 | 8E+06 | 8E+06 | 0.014 |
| **DG(17:1_18:2)** | DG | 1E+07 | 1E+07 | 6E+06 | 6E+06 | 2E+07 | 2E+07 | 2E+07 | 2E+07 | 0.015 |
| **DG(18:0_22:6)** | DG | 3E+08 | 2E+08 | 1E+08 | 1E+08 | 4E+08 | 3E+08 | 3E+08 | 3E+08 | 0.015 |
| **DG(18:2_20:4)** | DG | 3E+07 | 2E+07 | 2E+07 | 1E+07 | 5E+07 | 4E+07 | 9E+07 | 5E+07 | 0.016 |
| **DG(18:2_18:2)** | DG | 2E+09 | 2E+09 | 1E+09 | 1E+09 | 2E+09 | 2E+09 | 2E+09 | 2E+09 | 0.018 |
| **DG(16:1_22:6)** | DG | 1E+07 | 1E+07 | 6E+06 | 5E+06 | 2E+07 | 1E+07 | 2E+07 | 2E+07 | 0.018 |
| **DG(34:3e)** | DG | 2E+07 | 2E+07 | 2E+07 | 2E+07 | 4E+07 | 4E+07 | 3E+07 | 2E+07 | 0.018 |
| **DG(18:2_22:6)** | DG | 3E+06 | 3E+06 | 2E+06 | 5E+05 | 5E+06 | 4E+06 | 7E+06 | 3E+06 | 0.039 |
| **Hex1Cer(d18:1_16:0)** | Hex1Cer | 2E+07 | 1E+07 | 5E+06 | 5E+06 | 3E+07 | 2E+07 | 3E+07 | 2E+07 | 0.003 |
| **Hex1Cer(d18:1_22:0)** | Hex1Cer | 1E+07 | 1E+07 | 1E+07 | 9E+06 | 2E+07 | 2E+07 | 2E+07 | 1E+07 | 0.014 |
| **Hex2Cer(d18:1_22:0)** | Hex2Cer | 1E+07 | 1E+07 | 1E+07 | 1E+07 | 3E+07 | 2E+07 | 3E+07 | 2E+07 | 2E-04 |
| **Hex2Cer(d18:1_24:0)** | Hex2Cer | 1E+07 | 5E+06 | 3E+06 | 4E+06 | 2E+07 | 1E+07 | 2E+07 | 1E+07 | 0.01 |
| **LPC(15:0)** | LPC | 6E+06 | 6E+06 | 6E+06 | 6E+06 | 2E+07 | 2E+07 | 2E+07 | 2E+07 | 9E-07 |
| **LPC(16:1e)** | LPC | 1E+07 | 9E+06 | 9E+06 | 1E+07 | 2E+07 | 2E+07 | 2E+07 | 2E+07 | 2E-04 |
| **LPC(20:4e)** | LPC | 2E+07 | 1E+07 | 1E+07 | 1E+07 | 3E+07 | 4E+07 | 4E+07 | 3E+07 | 3E-04 |
| **LPC(16:1)** | LPC | 2E+06 | 1E+06 | 2E+06 | 2E+06 | 5E+06 | 4E+06 | 5E+06 | 4E+06 | 3E-04 |
| **LPC(16:0e)** | LPC | 5E+06 | 6E+06 | 5E+06 | 5E+06 | 1E+07 | 8E+06 | 1E+07 | 9E+06 | 0.002 |
| **LPC(24:1)** | LPC | 2E+07 | 6E+06 | 8E+06 | 8E+06 | 2E+07 | 4E+07 | 3E+07 | 2E+07 | 0.004 |
| **LPC(20:2)** | LPC | 6E+06 | 5E+05 | 4E+06 | 3E+06 | 8E+06 | 1E+07 | 1E+07 | 8E+06 | 0.006 |
| **LPC(20:3e)** | LPC | 8E+07 | 3E+07 | 5E+07 | 4E+07 | 9E+07 | 1E+08 | 1E+08 | 9E+07 | 0.007 |
| **LPC(18:2)** | LPC | 6E+06 | 2E+06 | 3E+06 | 3E+06 | 6E+06 | 9E+06 | 8E+06 | 6E+06 | 0.008 |
| **LPC(18:0)** | LPC | 4E+07 | 3E+07 | 4E+07 | 4E+07 | 5E+07 | 6E+07 | 6E+07 | 5E+07 | 0.012 |
| **LPC(18:1)** | LPC | 1E+07 | 4E+06 | 8E+06 | 7E+06 | 1E+07 | 2E+07 | 2E+07 | 1E+07 | 0.017 |
| **LPC(24:0)** | LPC | 3E+07 | 7E+06 | 3E+07 | 2E+07 | 4E+07 | 7E+07 | 7E+07 | 3E+07 | 0.017 |
| **LPC(22:0)** | LPC | 4E+07 | 2E+07 | 3E+07 | 3E+07 | 5E+07 | 8E+07 | 8E+07 | 4E+07 | 0.018 |
| **LPC(22:4)** | LPC | 3E+06 | 1E+06 | 2E+06 | 2E+06 | 3E+06 | 4E+06 | 4E+06 | 3E+06 | 0.02 |
| **LPC(19:0)** | LPC | 2E+06 | 1E+06 | 3E+06 | 2E+06 | 3E+06 | 3E+06 | 3E+06 | 3E+06 | 0.021 |
| **LPC(20:0)** | LPC | 3E+08 | 1E+08 | 3E+08 | 2E+08 | 4E+08 | 6E+08 | 6E+08 | 3E+08 | 0.023 |
| **LPC(18:3e)** | LPC | 1E+07 | 1E+07 | 9E+06 | 1E+07 | 2E+07 | 2E+07 | 2E+07 | 1E+07 | 0.026 |
| **LPC(22:3)** | LPC | 3E+06 | 5E+05 | 2E+06 | 1E+06 | 3E+06 | 4E+06 | 4E+06 | 3E+06 | 0.029 |
| **LPC(20:0e)** | LPC | 3E+06 | 2E+06 | 3E+06 | 2E+06 | 4E+06 | 5E+06 | 4E+06 | 3E+06 | 0.037 |
| **LPC(17:1)** | LPC | 4E+07 | 3E+07 | 3E+07 | 2E+07 | 4E+07 | 4E+07 | 5E+07 | 3E+07 | 0.045 |
| **LPE(20:0)** | LPE | 3E+06 | 1E+06 | 1E+06 | 2E+06 | 4E+06 | 6E+06 | 5E+06 | 4E+06 | 0.004 |
| **LPE(16:0)** | LPE | 4E+07 | 3E+07 | 3E+07 | 3E+07 | 3E+07 | 2E+07 | 2E+07 | 2E+07 | 0.007 |
| **LPE(18:0)** | LPE | 2E+06 | 2E+06 | 2E+06 | 1E+06 | 2E+06 | 2E+06 | 2E+06 | 3E+06 | 0.008 |
| **LPE(22:6)** | LPE | 8E+07 | 4E+07 | 7E+07 | 5E+07 | 8E+07 | 1E+08 | 1E+08 | 8E+07 | 0.02 |
| **LPE(18:0e)** | LPE | 5E+07 | 1E+07 | 3E+07 | 2E+07 | 5E+07 | 7E+07 | 7E+07 | 4E+07 | 0.021 |
| **LPE(20:1)** | LPE | 5E+06 | 4E+06 | 4E+06 | 4E+06 | 5E+06 | 5E+06 | 6E+06 | 7E+06 | 0.024 |
| **MePC(21:3e)** | MePC | 2E+07 | 6E+06 | 8E+06 | 7E+06 | 2E+07 | 4E+07 | 3E+07 | 2E+07 | 0.002 |
| **MePC(18:4e)** | MePC | 3E+07 | 1E+07 | 9E+06 | 8E+06 | 4E+07 | 5E+07 | 5E+07 | 4E+07 | 0.005 |
| **MePC(34:1)** | MePC | 2E+06 | 1E+06 | 2E+06 | 1E+06 | 3E+06 | 4E+06 | 4E+06 | 3E+06 | 0.008 |
| **MePC(36:5)** | MePC | 4E+06 | 3E+06 | 6E+06 | 5E+06 | 8E+06 | 9E+06 | 9E+06 | 6E+06 | 0.009 |
| **MePC(19:4e)** | MePC | 3E+06 | 1E+06 | 2E+06 | 5E+05 | 3E+06 | 4E+06 | 4E+06 | 3E+06 | 0.015 |
| **MePC(34:5e)** | MePC | 8E+06 | 6E+06 | 1E+07 | 4E+06 | 8E+06 | 2E+07 | 3E+07 | 2E+07 | 0.05 |
| **MG(18:0)** | MG | 4E+07 | 1E+07 | 2E+07 | 2E+07 | 6E+07 | 1E+08 | 9E+07 | 6E+07 | 0.003 |
| **PC(20:4_20:4)** | PC | 4E+07 | 5E+07 | 4E+07 | 4E+07 | 3E+07 | 3E+07 | 3E+07 | 3E+07 | 9E-04 |
| **PC(19:0_22:6)** | PC | 2E+07 | 2E+07 | 2E+07 | 2E+07 | 3E+07 | 3E+07 | 3E+07 | 2E+07 | 0.001 |
| **PC(16:0_22:6)** | PC | 1E+07 | 9E+06 | 9E+06 | 1E+07 | 1E+07 | 1E+07 | 2E+07 | 1E+07 | 0.003 |
| **PC(30:1)** | PC | 2E+06 | 5E+05 | 5E+05 | 2E+06 | 4E+06 | 7E+06 | 6E+06 | 4E+06 | 0.003 |
| **PC(42:1)** | PC | 4E+06 | 2E+06 | 2E+06 | 2E+06 | 5E+06 | 5E+06 | 5E+06 | 4E+06 | 0.004 |
| **PC(18:1_20:4)** | PC | 2E+06 | 1E+06 | 5E+05 | 3E+06 | 4E+06 | 4E+06 | 5E+06 | 5E+06 | 0.004 |
| **PC(42:3)** | PC | 9E+06 | 7E+06 | 7E+06 | 8E+06 | 1E+07 | 1E+07 | 1E+07 | 1E+07 | 0.005 |
| **PC(42:5)** | PC | 3E+07 | 3E+07 | 3E+07 | 3E+07 | 4E+07 | 4E+07 | 5E+07 | 3E+07 | 0.005 |
| **PC(18:0_18:2)** | PC | 1E+07 | 9E+06 | 8E+06 | 7E+06 | 1E+07 | 1E+07 | 2E+07 | 1E+07 | 0.005 |
| **PC(42:2)** | PC | 1E+07 | 1E+07 | 9E+06 | 9E+06 | 2E+07 | 2E+07 | 2E+07 | 1E+07 | 0.006 |
| **PC(16:1e_20:4)** | PC | 1E+07 | 7E+06 | 8E+06 | 5E+06 | 3E+07 | 4E+07 | 4E+07 | 2E+07 | 0.006 |
| **PC(20:3_18:2)** | PC | 3E+06 | 2E+06 | 2E+06 | 2E+06 | 5E+06 | 7E+06 | 7E+06 | 4E+06 | 0.006 |
| **PC(30:0)** | PC | 2E+06 | 1E+06 | 1E+06 | 5E+05 | 3E+06 | 5E+06 | 5E+06 | 3E+06 | 0.006 |
| **PC(34:0e)** | PC | 7E+08 | 4E+08 | 6E+08 | 5E+08 | 1E+09 | 9E+08 | 1E+09 | 8E+08 | 0.008 |
| **PC(19:0_20:4)** | PC | 500000 | 5E+05 | 5E+05 | 1E+06 | 1E+06 | 1E+06 | 1E+06 | 1E+06 | 0.009 |
| **PC(44:6)** | PC | 4E+09 | 4E+09 | 3E+09 | 3E+09 | 5E+09 | 7E+09 | 7E+09 | 5E+09 | 0.01 |
| **PC(14:0_20:4)** | PC | 6E+06 | 4E+06 | 6E+06 | 6E+06 | 9E+06 | 8E+06 | 1E+07 | 9E+06 | 0.011 |
| **PC(31:1)** | PC | 9E+07 | 7E+07 | 6E+07 | 7E+07 | 9E+07 | 9E+07 | 1E+08 | 1E+08 | 0.012 |
| **PC(41:7)** | PC | 2E+06 | 2E+06 | 3E+06 | 3E+06 | 5E+06 | 6E+06 | 7E+06 | 3E+06 | 0.012 |
| **PC(36:0)** | PC | 5E+08 | 3E+08 | 3E+08 | 3E+08 | 5E+08 | 6E+08 | 5E+08 | 5E+08 | 0.013 |
| **PC(19:3e)** | PC | 1E+06 | 5E+05 | 1E+06 | 5E+05 | 2E+06 | 2E+06 | 3E+06 | 1E+06 | 0.015 |
| **PC(35:4)** | PC | 1E+07 | 3E+06 | 5E+06 | 4E+06 | 1E+07 | 2E+07 | 2E+07 | 1E+07 | 0.016 |
| **PC(22:4_12:1)** | PC | 4E+07 | 2E+07 | 2E+07 | 2E+07 | 4E+07 | 4E+07 | 6E+07 | 4E+07 | 0.017 |
| **PC(39:5)** | PC | 5E+07 | 4E+07 | 4E+07 | 4E+07 | 7E+07 | 5E+07 | 7E+07 | 5E+07 | 0.018 |
| **PC(18:3e_22:1)** | PC | 2E+08 | 1E+08 | 1E+08 | 1E+08 | 2E+08 | 2E+08 | 2E+08 | 2E+08 | 0.02 |
| **PC(37:4e)** | PC | 2E+07 | 2E+07 | 2E+07 | 2E+07 | 2E+07 | 2E+07 | 3E+07 | 3E+07 | 0.023 |
| **PC(42:7)** | PC | 2E+07 | 1E+07 | 8E+06 | 8E+06 | 2E+07 | 3E+07 | 3E+07 | 2E+07 | 0.024 |
| **PC(38:2e)** | PC | 1E+08 | 6E+07 | 9E+07 | 8E+07 | 1E+08 | 1E+08 | 1E+08 | 1E+08 | 0.024 |
| **PC(44:7)** | PC | 2E+07 | 4E+06 | 2E+06 | 3E+06 | 2E+07 | 2E+07 | 2E+07 | 2E+07 | 0.025 |
| **PC(18:0_20:2)** | PC | 2E+07 | 2E+07 | 2E+07 | 2E+07 | 2E+07 | 3E+07 | 3E+07 | 2E+07 | 0.027 |
| **PC(16:0e_20:4)** | PC | 3E+06 | 4E+06 | 3E+06 | 3E+06 | 3E+06 | 1E+06 | 2E+06 | 2E+06 | 0.027 |
| **PC(18:1_22:6)** | PC | 2E+09 | 2E+09 | 2E+09 | 2E+09 | 3E+09 | 2E+09 | 3E+09 | 2E+09 | 0.027 |
| **PC(16:0_22:5)** | PC | 9E+06 | 2E+07 | 2E+07 | 2E+07 | 4E+07 | 3E+07 | 3E+07 | 2E+07 | 0.028 |
| **PC(33:2)** | PC | 2E+07 | 8E+06 | 8E+06 | 9E+06 | 2E+07 | 2E+07 | 2E+07 | 1E+07 | 0.029 |
| **PC(34:1e)** | PC | 4E+06 | 2E+06 | 1E+07 | 1E+07 | 2E+07 | 2E+07 | 3E+07 | 1E+07 | 0.031 |
| **PC(18:0_18:1)** | PC | 2E+07 | 1E+07 | 1E+07 | 1E+07 | 1E+07 | 5E+06 | 2E+06 | 1E+07 | 0.034 |
| **PC(18:0_20:4)** | PC | 1E+06 | 2E+06 | 2E+06 | 2E+06 | 2E+06 | 3E+06 | 3E+06 | 2E+06 | 0.035 |
| **PC(42:4)** | PC | 2E+07 | 2E+07 | 2E+07 | 2E+07 | 4E+07 | 4E+07 | 3E+07 | 2E+07 | 0.036 |
| **PC(38:6)** | PC | 4E+06 | 8E+06 | 6E+06 | 8E+06 | 8E+06 | 1E+07 | 9E+06 | 9E+06 | 0.038 |
| **PC(38:8)** | PC | 4E+08 | 3E+08 | 3E+08 | 3E+08 | 4E+08 | 5E+08 | 5E+08 | 4E+08 | 0.039 |
| **PC(40:7)** | PC | 9E+08 | 6E+08 | 6E+08 | 6E+08 | 1E+09 | 1E+09 | 1E+09 | 7E+08 | 0.041 |
| **PC(40:10)** | PC | 2E+08 | 1E+08 | 1E+08 | 1E+08 | 2E+08 | 2E+08 | 2E+08 | 2E+08 | 0.044 |
| **PC(42:8)** | PC | 1E+08 | 9E+07 | 8E+07 | 8E+07 | 1E+08 | 2E+08 | 1E+08 | 1E+08 | 0.045 |
| **PC(41:5)** | PC | 1E+07 | 9E+06 | 8E+06 | 8E+06 | 1E+07 | 1E+07 | 1E+07 | 1E+07 | 0.045 |
| **PC(17:1)** | PC | 1E+07 | 7E+06 | 2E+07 | 8E+06 | 2E+07 | 2E+07 | 3E+07 | 2E+07 | 0.049 |
| **PE(20:0p_20:4)** | PE | 3E+08 | 3E+08 | 3E+08 | 3E+08 | 4E+08 | 4E+08 | 4E+08 | 4E+08 | 2E-04 |
| **PE(16:1e_22:6)** | PE | 5E+06 | 5E+06 | 5E+06 | 4E+06 | 6E+06 | 6E+06 | 7E+06 | 6E+06 | 2E-04 |
| **PE(16:1_22:6)** | PE | 2E+06 | 1E+06 | 1E+06 | 1E+06 | 3E+06 | 4E+06 | 4E+06 | 3E+06 | 4E-04 |
| **PE(20:1e_20:4)** | PE | 1E+08 | 4E+07 | 6E+07 | 6E+07 | 1E+08 | 2E+08 | 2E+08 | 2E+08 | 0.001 |
| **PE(36:1e)** | PE | 5E+06 | 2E+06 | 3E+06 | 4E+06 | 1E+07 | 1E+07 | 1E+07 | 8E+06 | 0.001 |
| **PE(20:0e_22:6)** | PE | 3E+08 | 1E+08 | 2E+08 | 2E+08 | 4E+08 | 6E+08 | 4E+08 | 4E+08 | 0.001 |
| **PE(17:0_18:1)** | PE | 8E+07 | 4E+07 | 7E+07 | 7E+07 | 1E+08 | 2E+08 | 2E+08 | 1E+08 | 0.002 |
| **PE(20:2_22:6)** | PE | 9E+06 | 8E+06 | 7E+06 | 8E+06 | 1E+07 | 1E+07 | 1E+07 | 1E+07 | 0.002 |
| **PE(14:0_22:6)** | PE | 4E+06 | 2E+06 | 3E+06 | 3E+06 | 7E+06 | 1E+07 | 9E+06 | 6E+06 | 0.002 |
| **PE(18:0_16:0)** | PE | 7E+07 | 6E+07 | 4E+07 | 4E+07 | 1E+08 | 1E+08 | 1E+08 | 9E+07 | 0.002 |
| **PE(20:2e)** | PE | 2E+08 | 2E+08 | 2E+08 | 2E+08 | 3E+08 | 4E+08 | 4E+08 | 3E+08 | 0.002 |
| **PE(20:0e_20:4)** | PE | 4E+06 | 2E+06 | 2E+06 | 2E+06 | 6E+06 | 9E+06 | 9E+06 | 6E+06 | 0.002 |
| **PE(16:0_22:6)** | PE | 6E+06 | 2E+06 | 4E+06 | 2E+06 | 8E+06 | 1E+07 | 1E+07 | 9E+06 | 0.002 |
| **PE(16:0_20:1)** | PE | 4E+06 | 3E+06 | 5E+06 | 4E+06 | 7E+06 | 7E+06 | 8E+06 | 7E+06 | 0.002 |
| **PE(18:1e_22:6)** | PE | 2E+07 | 8E+06 | 1E+07 | 1E+07 | 3E+07 | 5E+07 | 5E+07 | 3E+07 | 0.003 |
| **PE(18:0_22:4)** | PE | 4E+06 | 2E+06 | 3E+06 | 2E+06 | 6E+06 | 5E+06 | 8E+06 | 6E+06 | 0.003 |
| **PE(15:0_18:2)** | PE | 3E+08 | 3E+08 | 3E+08 | 2E+08 | 3E+08 | 3E+08 | 3E+08 | 3E+08 | 0.003 |
| **PE(18:0e_22:6)** | PE | 2E+06 | 5E+05 | 1E+06 | 5E+05 | 3E+06 | 4E+06 | 4E+06 | 2E+06 | 0.004 |
| **PE(16:0_18:1)** | PE | 7E+06 | 6E+06 | 5E+06 | 5E+06 | 1E+07 | 1E+07 | 1E+07 | 8E+06 | 0.004 |
| **PE(20:3_22:6)** | PE | 1E+08 | 1E+08 | 1E+08 | 1E+08 | 2E+08 | 2E+08 | 3E+08 | 2E+08 | 0.004 |
| **PE(18:3_18:2)** | PE | 5E+08 | 2E+08 | 3E+08 | 2E+08 | 6E+08 | 1E+09 | 8E+08 | 6E+08 | 0.004 |
| **PE(18:2e_22:6)** | PE | 1E+06 | 5E+05 | 5E+05 | 5E+05 | 2E+06 | 4E+06 | 4E+06 | 2E+06 | 0.005 |
| **PE(19:0_22:6)** | PE | 2E+06 | 5E+05 | 1E+06 | 1E+06 | 3E+06 | 5E+06 | 5E+06 | 3E+06 | 0.005 |
| **PE(20:1_20:4)** | PE | 5E+07 | 2E+07 | 5E+07 | 4E+07 | 8E+07 | 1E+08 | 1E+08 | 6E+07 | 0.008 |
| **PE(19:0_20:4)** | PE | 9E+07 | 6E+07 | 6E+07 | 7E+07 | 9E+07 | 9E+07 | 1E+08 | 1E+08 | 0.009 |
| **PE(18:0p_22:5)** | PE | 500000 | 5E+05 | 5E+05 | 1E+06 | 1E+06 | 3E+06 | 3E+06 | 2E+06 | 0.009 |
| **PE(36:2e)** | PE | 8E+06 | 7E+06 | 5E+06 | 4E+06 | 9E+06 | 1E+07 | 1E+07 | 9E+06 | 0.01 |
| **PE(17:1_20:4)** | PE | 1E+07 | 5E+06 | 3E+06 | 4E+06 | 2E+07 | 1E+07 | 2E+07 | 1E+07 | 0.01 |
| **PE(17:1_18:2)** | PE | 1E+07 | 6E+06 | 8E+06 | 8E+06 | 1E+07 | 1E+07 | 1E+07 | 1E+07 | 0.011 |
| **PE(37:1)** | PE | 2E+08 | 2E+08 | 2E+08 | 2E+08 | 3E+08 | 2E+08 | 3E+08 | 3E+08 | 0.011 |
| **PE(18:0_22:6)** | PE | 3E+06 | 2E+06 | 3E+06 | 3E+06 | 4E+06 | 5E+06 | 6E+06 | 3E+06 | 0.012 |
| **PE(15:0_20:4)** | PE | 6E+06 | 5E+06 | 4E+06 | 5E+06 | 9E+06 | 9E+06 | 9E+06 | 6E+06 | 0.013 |
| **PE(18:0e_20:4)** | PE | 1E+07 | 1E+07 | 8E+06 | 1E+07 | 2E+07 | 2E+07 | 2E+07 | 1E+07 | 0.013 |
| **PE(18:0_22:2)** | PE | 3E+08 | 3E+08 | 2E+08 | 2E+08 | 4E+08 | 5E+08 | 6E+08 | 4E+08 | 0.013 |
| **PE(16:0_20:4)** | PE | 2E+06 | 2E+06 | 2E+06 | 2E+06 | 4E+06 | 4E+06 | 3E+06 | 2E+06 | 0.013 |
| **PE(37:2)** | PE | 2E+08 | 3E+08 | 2E+08 | 2E+08 | 3E+08 | 3E+08 | 3E+08 | 3E+08 | 0.013 |
| **PE(16:1_18:2)** | PE | 5E+07 | 4E+07 | 4E+07 | 4E+07 | 6E+07 | 8E+07 | 8E+07 | 5E+07 | 0.014 |
| **PE(14:0_18:2)** | PE | 1E+09 | 7E+08 | 8E+08 | 8E+08 | 1E+09 | 1E+09 | 2E+09 | 1E+09 | 0.015 |
| **PE(18:1p_18:2)** | PE | 2E+06 | 2E+06 | 3E+06 | 2E+06 | 4E+06 | 7E+06 | 6E+06 | 3E+06 | 0.015 |
| **PE(18:0_18:0)** | PE | 2E+08 | 2E+08 | 2E+08 | 2E+08 | 3E+08 | 3E+08 | 3E+08 | 2E+08 | 0.017 |
| **PE(39:2e)** | PE | 4E+07 | 4E+07 | 3E+07 | 3E+07 | 5E+07 | 6E+07 | 7E+07 | 4E+07 | 0.017 |
| **PE(33:10)** | PE | 2E+07 | 2E+07 | 2E+07 | 2E+07 | 3E+07 | 3E+07 | 3E+07 | 2E+07 | 0.019 |
| **PE(43:6)** | PE | 3E+07 | 2E+07 | 3E+07 | 3E+07 | 3E+07 | 3E+07 | 4E+07 | 4E+07 | 0.02 |
| **PE(20:3_18:2)** | PE | 1E+07 | 2E+07 | 1E+07 | 1E+07 | 2E+07 | 2E+07 | 2E+07 | 2E+07 | 0.021 |
| **PE(22:0_18:2)** | PE | 7E+07 | 6E+07 | 4E+07 | 5E+07 | 7E+07 | 8E+07 | 9E+07 | 7E+07 | 0.022 |
| **PE(17:0_20:4)** | PE | 2E+08 | 2E+08 | 2E+08 | 2E+08 | 3E+08 | 3E+08 | 3E+08 | 2E+08 | 0.023 |
| **PE(20:0_20:4)** | PE | 6E+07 | 3E+07 | 5E+07 | 4E+07 | 7E+07 | 8E+07 | 9E+07 | 6E+07 | 0.026 |
| **PE(52:1)** | PE | 3E+07 | 3E+07 | 3E+07 | 3E+07 | 4E+07 | 5E+07 | 4E+07 | 3E+07 | 0.026 |
| **PE(22:5e)** | PE | 3E+07 | 2E+07 | 2E+07 | 2E+07 | 3E+07 | 3E+07 | 4E+07 | 3E+07 | 0.028 |
| **PE(18:0_22:5)** | PE | 7E+06 | 2E+06 | 7E+06 | 5E+06 | 9E+06 | 1E+07 | 1E+07 | 7E+06 | 0.029 |
| **PE(32:1e)** | PE | 1E+08 | 1E+08 | 1E+08 | 1E+08 | 8E+07 | 9E+07 | 8E+07 | 9E+07 | 0.03 |
| **PE(20:0_18:2)** | PE | 4E+06 | 1E+06 | 3E+06 | 2E+06 | 4E+06 | 6E+06 | 6E+06 | 4E+06 | 0.03 |
| **PE(16:1e_22:4)** | PE | 2E+06 | 2E+06 | 2E+06 | 2E+06 | 3E+06 | 3E+06 | 3E+06 | 2E+06 | 0.033 |
| **PE(39:2)** | PE | 7E+06 | 7E+06 | 4E+06 | 5E+06 | 8E+06 | 7E+06 | 8E+06 | 9E+06 | 0.033 |
| **PE(37:0)** | PE | 7E+07 | 4E+07 | 5E+07 | 5E+07 | 7E+07 | 9E+07 | 8E+07 | 6E+07 | 0.036 |
| **PE(16:1_20:4)** | PE | 2E+07 | 8E+06 | 3E+07 | 1E+07 | 3E+07 | 5E+07 | 4E+07 | 3E+07 | 0.037 |
| **PE(39:3)** | PE | 7E+07 | 4E+07 | 5E+07 | 5E+07 | 7E+07 | 8E+07 | 8E+07 | 6E+07 | 0.037 |
| **PE(18:0_20:5)** | PE | 9E+06 | 6E+06 | 6E+06 | 6E+06 | 1E+07 | 1E+07 | 1E+07 | 8E+06 | 0.037 |
| **PE(43:3)** | PE | 3E+07 | 2E+07 | 2E+07 | 2E+07 | 3E+07 | 3E+07 | 3E+07 | 3E+07 | 0.037 |
| **PE(16:0_16:1)** | PE | 1E+07 | 8E+06 | 6E+06 | 7E+06 | 1E+07 | 1E+07 | 1E+07 | 9E+06 | 0.04 |
| **PE(14:0_20:4)** | PE | 9E+07 | 8E+07 | 6E+07 | 7E+07 | 9E+07 | 1E+08 | 1E+08 | 8E+07 | 0.04 |
| **PE(18:0p_22:4)** | PE | 2E+08 | 2E+08 | 2E+08 | 2E+08 | 3E+08 | 2E+08 | 2E+08 | 2E+08 | 0.04 |
| **PE(18:1_18:3)** | PE | 3E+06 | 1E+06 | 2E+06 | 2E+06 | 3E+06 | 4E+06 | 4E+06 | 3E+06 | 0.042 |
| **PE(18:1e_20:4)** | PE | 2E+07 | 2E+07 | 2E+07 | 2E+07 | 2E+07 | 2E+07 | 2E+07 | 2E+07 | 0.043 |
| **PE(15:0_22:6)** | PE | 7E+06 | 5E+06 | 8E+06 | 7E+06 | 8E+06 | 9E+06 | 9E+06 | 8E+06 | 0.044 |
| **PE(20:3_20:4)** | PE | 1E+08 | 6E+07 | 1E+08 | 8E+07 | 2E+08 | 2E+08 | 2E+08 | 1E+08 | 0.044 |
| **PE(18:0_20:1)** | PE | 2E+09 | 2E+09 | 2E+09 | 2E+09 | 2E+09 | 2E+09 | 2E+09 | 2E+09 | 0.045 |
| **PE(16:0_18:2)** | PE | 5E+06 | 4E+06 | 5E+06 | 4E+06 | 7E+06 | 7E+06 | 7E+06 | 5E+06 | 0.045 |
| **PE(22:1_20:4)** | PE | 2E+08 | 1E+08 | 1E+08 | 1E+08 | 2E+08 | 2E+08 | 2E+08 | 1E+08 | 0.048 |
| **PE(20:5e)** | PE | 7E+08 | 8E+08 | 6E+08 | 6E+08 | 8E+08 | 9E+08 | 9E+08 | 7E+08 | 0.049 |
| **PE(18:3_22:6)** | PE | 500000 | 1E+07 | 1E+07 | 1E+07 | 1E+07 | 1E+07 | 2E+07 | 2E+07 | 0.05 |
| **PG(16:0_20:1)** | PG | 3E+06 | 1E+06 | 2E+06 | 2E+06 | 4E+06 | 6E+06 | 6E+06 | 4E+06 | 0.001 |
| **PG(18:2_22:6)** | PG | 2E+06 | 1E+06 | 2E+06 | 2E+06 | 4E+06 | 4E+06 | 4E+06 | 3E+06 | 0.002 |
| **PG(22:5_22:6)** | PG | 4E+06 | 2E+06 | 2E+06 | 2E+06 | 6E+06 | 9E+06 | 9E+06 | 6E+06 | 0.002 |
| **PG(20:3_22:6)** | PG | 2E+07 | 8E+06 | 1E+07 | 1E+07 | 3E+07 | 5E+07 | 5E+07 | 3E+07 | 0.003 |
| **PG(16:0_18:2)** | PG | 3E+06 | 2E+06 | 2E+06 | 2E+06 | 4E+06 | 5E+06 | 5E+06 | 4E+06 | 0.003 |
| **PG(22:4_22:6)** | PG | 1E+06 | 5E+05 | 5E+05 | 5E+05 | 2E+06 | 2E+06 | 2E+06 | 2E+06 | 0.003 |
| **PG(18:1_20:4)** | PG | 6E+07 | 4E+07 | 6E+07 | 5E+07 | 8E+07 | 8E+07 | 8E+07 | 7E+07 | 0.004 |
| **PG(22:5_20:4)** | PG | 2E+06 | 5E+05 | 1E+06 | 5E+05 | 3E+06 | 4E+06 | 4E+06 | 2E+06 | 0.004 |
| **PG(22:6_22:6)** | PG | 6E+06 | 4E+06 | 6E+06 | 5E+06 | 9E+06 | 9E+06 | 1E+07 | 7E+06 | 0.005 |
| **PG(20:4_22:6)** | PG | 1E+08 | 4E+07 | 1E+08 | 7E+07 | 2E+08 | 3E+08 | 2E+08 | 2E+08 | 0.005 |
| **PG(18:3_18:2)** | PG | 1E+08 | 2E+08 | 2E+08 | 2E+08 | 3E+08 | 3E+08 | 3E+08 | 2E+08 | 0.007 |
| **PG(22:5_18:2)** | PG | 2E+08 | 8E+07 | 2E+08 | 1E+08 | 3E+08 | 4E+08 | 5E+08 | 3E+08 | 0.007 |
| **PG(18:1_18:2)** | PG | 500000 | 5E+05 | 1E+06 | 5E+05 | 1E+06 | 2E+06 | 2E+06 | 1E+06 | 0.009 |
| **PG(18:1_18:1)** | PG | 2E+06 | 5E+05 | 5E+05 | 5E+05 | 2E+06 | 2E+06 | 2E+06 | 1E+06 | 0.015 |
| **PG(22:4_20:4)** | PG | 1E+06 | 5E+05 | 5E+05 | 5E+05 | 2E+06 | 3E+06 | 3E+06 | 2E+06 | 0.016 |
| **PG(16:1_22:6)** | PG | 4E+07 | 3E+07 | 3E+07 | 3E+07 | 5E+07 | 5E+07 | 5E+07 | 4E+07 | 0.027 |
| **PG(18:1_22:5)** | PG | 4E+07 | 1E+07 | 3E+07 | 3E+07 | 4E+07 | 5E+07 | 6E+07 | 5E+07 | 0.029 |
| **PG(18:1_22:6)** | PG | 3E+07 | 9E+06 | 3E+07 | 2E+07 | 4E+07 | 7E+07 | 6E+07 | 3E+07 | 0.033 |
| **PG(18:2_18:2)** | PG | 2E+06 | 1E+06 | 2E+06 | 1E+06 | 4E+06 | 2E+06 | 4E+06 | 2E+06 | 0.049 |
| **PI(20:0_20:4)** | PI | 9E+06 | 9E+06 | 9E+06 | 9E+06 | 1E+07 | 1E+07 | 1E+07 | 1E+07 | 6E-04 |
| **PI(18:1_20:4)** | PI | 1E+07 | 1E+07 | 1E+07 | 1E+07 | 2E+07 | 2E+07 | 2E+07 | 2E+07 | 0.001 |
| **PI(18:0_20:5)** | PI | 5E+06 | 4E+06 | 4E+06 | 5E+06 | 6E+06 | 6E+06 | 6E+06 | 5E+06 | 0.003 |
| **PI(18:0_20:3)** | PI | 1E+07 | 6E+06 | 6E+06 | 7E+06 | 1E+07 | 1E+07 | 1E+07 | 1E+07 | 0.005 |
| **PI(18:0_18:1)** | PI | 2E+07 | 2E+07 | 2E+07 | 2E+07 | 2E+07 | 2E+07 | 3E+07 | 2E+07 | 0.006 |
| **PI(18:0_22:6)** | PI | 3E+07 | 2E+07 | 2E+07 | 2E+07 | 3E+07 | 4E+07 | 4E+07 | 3E+07 | 0.012 |
| **PI(18:0_22:5)** | PI | 3E+06 | 3E+06 | 3E+06 | 3E+06 | 3E+06 | 3E+06 | 4E+06 | 3E+06 | 0.014 |
| **PI(18:0_22:4)** | PI | 1E+07 | 7E+06 | 1E+07 | 8E+06 | 1E+07 | 1E+07 | 1E+07 | 1E+07 | 0.018 |
| **PI(16:0_20:4)** | PI | 3E+07 | 4E+07 | 5E+07 | 4E+07 | 7E+07 | 6E+07 | 7E+07 | 4E+07 | 0.032 |
| **PS(39:6)** | PS | 1E+07 | 1E+07 | 1E+07 | 1E+07 | 2E+07 | 2E+07 | 2E+07 | 1E+07 | 0.005 |
| **PS(42:9e)** | PS | 3E+06 | 2E+06 | 2E+06 | 1E+06 | 5E+06 | 5E+06 | 8E+06 | 4E+06 | 0.007 |
| **PS(20:4_20:4)** | PS | 4E+06 | 4E+06 | 3E+06 | 3E+06 | 6E+06 | 6E+06 | 6E+06 | 4E+06 | 0.012 |
| **PS(18:0_22:6)** | PS | 8E+06 | 6E+06 | 5E+06 | 7E+06 | 9E+06 | 9E+06 | 9E+06 | 8E+06 | 0.021 |
| **PS(18:0_18:2)** | PS | 4E+07 | 3E+07 | 3E+07 | 3E+07 | 8E+07 | 8E+07 | 5E+07 | 5E+07 | 0.021 |
| **PS(18:0_20:3)** | PS | 1E+07 | 8E+06 | 6E+06 | 6E+06 | 1E+07 | 1E+07 | 1E+07 | 9E+06 | 0.022 |
| **PS(42:6)** | PS | 8E+08 | 6E+08 | 7E+08 | 8E+08 | 9E+08 | 9E+08 | 1E+09 | 8E+08 | 0.026 |
| **PS(19:0_20:4)** | PS | 4E+06 | 5E+06 | 5E+06 | 4E+06 | 6E+06 | 6E+06 | 7E+06 | 5E+06 | 0.028 |
| **PS(40:7)** | PS | 3E+07 | 2E+07 | 2E+07 | 2E+07 | 3E+07 | 3E+07 | 3E+07 | 3E+07 | 0.034 |
| **SM(d18:1_24:4)** | SM | 2E+06 | 3E+06 | 5E+06 | 4E+06 | 7E+06 | 6E+06 | 6E+06 | 5E+06 | 0.006 |
| **SM(d42:4)** | SM | 500000 | 1E+06 | 1E+06 | 5E+05 | 2E+06 | 2E+06 | 2E+06 | 2E+06 | 0.007 |
| **SM(t18:1_24:2)** | SM | 4E+07 | 4E+07 | 6E+07 | 5E+07 | 8E+07 | 7E+07 | 8E+07 | 5E+07 | 0.027 |
| **SM(t42:1)** | SM | 1E+07 | 6E+06 | 7E+06 | 6E+06 | 8E+06 | 1E+07 | 1E+07 | 1E+07 | 0.036 |
| **SM(d18:1_24:3)** | SM | 1E+08 | 1E+08 | 1E+08 | 1E+08 | 2E+08 | 2E+08 | 2E+08 | 1E+08 | 0.037 |
| **SPH(d18:0)** | SPH | 1E+07 | 1E+07 | 1E+07 | 1E+07 | 2E+07 | 2E+07 | 2E+07 | 2E+07 | 0.002 |
| **SPH(d24:0)** | SPH | 4E+06 | 3E+06 | 4E+06 | 4E+06 | 7E+06 | 7E+06 | 8E+06 | 5E+06 | 0.003 |
| **SPH(t18:0)** | SPH | 6E+06 | 5E+06 | 8E+06 | 7E+06 | 1E+07 | 1E+07 | 9E+06 | 9E+06 | 0.012 |
| **TG(16:0_12:0_22:6)** | TG | 1E+08 | 9E+07 | 1E+08 | 1E+08 | 2E+08 | 2E+08 | 2E+08 | 2E+08 | 3E-04 |
| **TG(8:0_12:0_14:0)** | TG | 7E+06 | 4E+06 | 5E+06 | 6E+06 | 1E+07 | 1E+07 | 1E+07 | 1E+07 | 5E-04 |
| **TG(16:0_18:1_22:0)** | TG | 1E+07 | 1E+07 | 1E+07 | 1E+07 | 7E+06 | 2E+06 | 2E+06 | 3E+06 | 6E-04 |
| **TG(18:1_18:1_24:0)** | TG | 8E+07 | 4E+07 | 5E+07 | 5E+07 | 1E+08 | 1E+08 | 1E+08 | 1E+08 | 7E-04 |
| **TG(18:0e_18:2_18:2)** | TG | 2E+07 | 1E+07 | 1E+07 | 1E+07 | 4E+07 | 3E+07 | 4E+07 | 3E+07 | 0.001 |
| **TG(16:0_10:0_18:2)** | TG | 7E+07 | 6E+07 | 7E+07 | 6E+07 | 9E+07 | 1E+08 | 1E+08 | 8E+07 | 0.001 |
| **TG(8:0_12:0_12:0)** | TG | 1E+08 | 7E+07 | 1E+08 | 8E+07 | 2E+08 | 2E+08 | 2E+08 | 2E+08 | 0.001 |
| **TG(22:5_18:2_22:6)** | TG | 7E+06 | 5E+06 | 6E+06 | 6E+06 | 9E+06 | 1E+07 | 1E+07 | 9E+06 | 0.002 |
| **TG(16:0_18:1_20:4)** | TG | 3E+07 | 3E+07 | 2E+07 | 2E+07 | 1E+07 | 4E+06 | 4E+06 | 7E+06 | 0.003 |
| **TG(18:1_18:2_22:4)** | TG | 7E+06 | 4E+06 | 5E+06 | 5E+06 | 1E+07 | 1E+07 | 1E+07 | 8E+06 | 0.003 |
| **TG(18:1_18:2_18:2)** | TG | 3E+06 | 3E+06 | 2E+06 | 2E+06 | 2E+06 | 5E+05 | 1E+06 | 1E+06 | 0.004 |
| **TG(18:1e_16:0_16:0)** | TG | 1E+06 | 1E+06 | 5E+05 | 5E+05 | 2E+06 | 2E+06 | 3E+06 | 2E+06 | 0.004 |
| **TG(18:1_18:2_24:1)** | TG | 1E+07 | 6E+06 | 1E+07 | 1E+07 | 2E+07 | 2E+07 | 2E+07 | 2E+07 | 0.004 |
| **TG(16:0_10:2_13:0)** | TG | 7E+07 | 5E+07 | 7E+07 | 6E+07 | 9E+07 | 1E+08 | 1E+08 | 9E+07 | 0.004 |
| **TG(16:0_18:1_22:6)** | TG | 1E+07 | 1E+07 | 1E+07 | 1E+07 | 7E+06 | 2E+06 | 4E+06 | 8E+06 | 0.005 |
| **TG(15:0_18:2_18:3)** | TG | 2E+09 | 1E+09 | 2E+09 | 2E+09 | 3E+09 | 3E+09 | 3E+09 | 3E+09 | 0.005 |
| **TG(16:0_18:2_18:2)** | TG | 3E+06 | 2E+06 | 3E+06 | 3E+06 | 6E+06 | 6E+06 | 5E+06 | 4E+06 | 0.005 |
| **TG(16:1_16:1_18:2)** | TG | 7E+08 | 5E+08 | 6E+08 | 7E+08 | 9E+08 | 8E+08 | 9E+08 | 8E+08 | 0.005 |
| **TG(18:1_18:2_20:4)** | TG | 2E+07 | 1E+07 | 1E+07 | 1E+07 | 1E+07 | 7E+06 | 6E+06 | 6E+06 | 0.006 |
| **TG(18:3e_9:0_9:0)** | TG | 5E+06 | 3E+06 | 5E+06 | 4E+06 | 6E+06 | 9E+06 | 1E+07 | 8E+06 | 0.007 |
| **TG(16:0_16:1_18:1)** | TG | 6E+06 | 6E+06 | 6E+06 | 6E+06 | 7E+06 | 7E+06 | 8E+06 | 9E+06 | 0.008 |
| **TG(19:1_18:2_18:2)** | TG | 3E+07 | 4E+07 | 3E+07 | 3E+07 | 3E+07 | 9E+06 | 1E+07 | 2E+07 | 0.012 |
| **TG(18:0_18:0_18:0)** | TG | 9E+06 | 1E+07 | 6E+06 | 6E+06 | 5E+06 | 2E+06 | 1E+06 | 2E+06 | 0.012 |
| **TG(16:0_17:0_18:1)** | TG | 2E+07 | 1E+07 | 1E+07 | 1E+07 | 3E+07 | 2E+07 | 2E+07 | 2E+07 | 0.023 |
| **TG(16:0_20:4_22:0)** | TG | 6E+06 | 1E+07 | 1E+07 | 9E+06 | 5E+06 | 5E+06 | 5E+06 | 5E+05 | 0.023 |
| **TG(16:1_18:3_18:3)** | TG | 1E+07 | 1E+07 | 1E+07 | 1E+07 | 2E+07 | 2E+07 | 2E+07 | 1E+07 | 0.024 |
| **TG(15:0_18:2_18:2)** | TG | 4E+07 | 2E+07 | 2E+07 | 2E+07 | 5E+07 | 3E+07 | 4E+07 | 4E+07 | 0.026 |
| **TG(12:0_14:0_14:0)** | TG | 2E+08 | 1E+08 | 1E+08 | 1E+08 | 2E+08 | 2E+08 | 2E+08 | 2E+08 | 0.026 |
| **TG(17:0_18:2_20:4)** | TG | 2E+07 | 2E+07 | 2E+07 | 2E+07 | 2E+07 | 3E+06 | 3E+06 | 5E+06 | 0.027 |
| **TG(16:0_12:0_14:0)** | TG | 4E+06 | 2E+06 | 2E+06 | 2E+06 | 5E+06 | 4E+06 | 5E+06 | 3E+06 | 0.028 |
| **TG(12:0_12:0_14:0)** | TG | 3E+06 | 3E+06 | 2E+06 | 2E+06 | 5E+06 | 4E+06 | 5E+06 | 3E+06 | 0.033 |
| **TG(16:0_8:0_20:4)** | TG | 3E+09 | 2E+09 | 2E+09 | 2E+09 | 3E+09 | 3E+09 | 3E+09 | 2E+09 | 0.033 |
| **TG(44:6e)** | TG | 5E+06 | 3E+06 | 4E+06 | 2E+06 | 7E+06 | 5E+06 | 8E+06 | 4E+06 | 0.034 |
| **TG(16:0_18:1_22:1)** | TG | 2E+06 | 1E+06 | 2E+06 | 2E+06 | 3E+06 | 2E+06 | 3E+06 | 3E+06 | 0.036 |
| **TG(20:0_16:0_18:1)** | TG | 1E+07 | 1E+07 | 6E+06 | 8E+06 | 9E+06 | 3E+06 | 2E+06 | 2E+06 | 0.043 |
| **TG(16:0_14:0_18:1)** | TG | 8E+06 | 5E+06 | 8E+06 | 4E+06 | 9E+06 | 9E+06 | 1E+07 | 8E+06 | 0.043 |
| **TG(15:0_18:1_18:2)** | TG | 1E+07 | 9E+06 | 8E+06 | 7E+06 | 1E+07 | 1E+07 | 1E+07 | 1E+07 | 0.044 |
| **TG(18:0_16:0_17:0)** | TG | 1E+08 | 1E+08 | 9E+07 | 9E+07 | 1E+08 | 1E+08 | 1E+08 | 1E+08 | 0.045 |
| **TG(16:0_10:3_18:1)** | TG | 3E+06 | 3E+06 | 2E+06 | 2E+06 | 3E+06 | 3E+06 | 5E+06 | 4E+06 | 0.045 |
| **TG(18:2_18:2_18:2)** | TG | 4E+06 | 4E+06 | 2E+06 | 2E+06 | 4E+06 | 4E+06 | 5E+06 | 4E+06 | 0.049 |

**Supplemental Table S2.** Targeted lipidomics on liver homogenates and mitochondrial fractions from Alb-Cre and SPT3-hKO murine samples.

|  | **Liver Homogenate** | | | | | | | | **Mitochondrial Fraction** | | | | | | |
| --- | --- | --- | --- | --- | --- | --- | --- | --- | --- | --- | --- | --- | --- | --- | --- |
|  | **d16 Sphingolipids Normalized values** | | | | | | | **t-test** | **d16 Sphingolipids Normalized values** | | | | | | **t-test** |
| **Species** | **Alb-Cre** | | | **SPT3-hKO** | | | | **KO vs WT** | **Alb-Cre** | | | **SPT3-hKO** | | | **KO vs WT** |
| d16:1 Sph | 0.08 | 0.10 | 0.10 | 0.07 | 0.06 | 0.03 | 0.04 | 0.025 | 0.02 | 0.10 | 0.05 | 0.02 | 0.04 | 0.04 | 0.347 |
| d16:0 DHS | 1.46 | 1.65 | 1.76 | 1.56 | 1.83 | 1.77 | 1.34 | 0.992 | 0.00 | 0.00 | 0.00 | 0.00 | 0.00 | 0.00 | N/A |
| d16:1 S1P | 0.03 | 0.03 | 0.03 | 0.02 | 0.04 | 0.03 | 0.03 | 0.833 | 0.03 | 0.02 | 0.05 | 0.01 | 0.05 | 0.01 | 0.562 |
| d16:0 DHS1P | 0.12 | 0.22 | 0.21 | 0.08 | 0.11 | 0.10 | 0.07 | 0.019 | 0.03 | 0.17 | 0.09 | 0.08 | 0.04 | 0.04 | 0.375 |
| C14 Cer | 0.59 | 0.72 | 0.54 | 0.95 | 0.66 | 0.75 | 0.74 | 0.119 | 0.53 | 0.54 | 0.52 | 0.55 | 0.46 | 0.57 | 0.890 |
| C16 Cer | 0.54 | 0.52 | 0.71 | 0.65 | 0.60 | 0.55 | 0.50 | 0.837 | 0.85 | 0.94 | 0.79 | 0.30 | 0.58 | 0.55 | 0.017 |
| C18:1 Cer | 0.00 | 0.00 | 0.01 | 0.01 | 0.00 | 0.01 | 0.01 | 0.268 | 0.00 | 0.00 | 0.00 | 0.00 | 0.00 | 0.00 | N/A |
| C18:0 Cer | 0.02 | 0.02 | 0.04 | 0.03 | 0.02 | 0.03 | 0.03 | 0.929 | 0.04 | 0.05 | 0.01 | 0.02 | 0.02 | 0.04 | 0.595 |
| C20 Cer | 0.05 | 0.05 | 0.04 | 0.05 | 0.03 | 0.04 | 0.04 | 0.152 | 0.08 | 0.07 | 0.04 | 0.03 | 0.03 | 0.03 | 0.062 |
| C22 Cer | 4.18 | 3.88 | 4.29 | 4.65 | 3.85 | 3.74 | 4.05 | 0.867 | 7.43 | 7.80 | 5.14 | 3.20 | 4.47 | 3.60 | 0.029 |
| C24:1 Cer | 2.06 | 1.87 | 2.34 | 2.17 | 2.14 | 2.48 | 2.14 | 0.396 | 10.82 | 13.27 | 9.26 | 4.49 | 5.61 | 4.84 | 0.007 |
| C24:0 Cer | 2.08 | 2.74 | 2.37 | 3.35 | 2.24 | 2.10 | 2.49 | 0.706 | 6.39 | 6.85 | 5.21 | 2.04 | 4.24 | 4.00 | 0.033 |
| C26:1 Cer | 1.00 | 0.82 | 1.20 | 1.49 | 1.07 | 1.08 | 1.16 | 0.254 | 4.12 | 5.09 | 3.77 | 1.24 | 2.46 | 2.36 | 0.014 |
| C26:0 Cer | 1.22 | 1.09 | 1.70 | 1.97 | 1.81 | 1.61 | 1.64 | 0.069 | 3.88 | 4.67 | 3.47 | 2.05 | 3.25 | 2.49 | 0.047 |
|  | **d17 Sphingolipids Normalized values** | | | | | | | **t-test** | **d17 Sphingolipids Normalized values** | | | | | | **t-test** |
| **Species** | **Alb-Cre** | | | **SPT3-hKO** | | | | **KO vs WT** | **Alb-Cre** | | | **SPT3-hKO** | | | **KO vs WT** |
| C14 Cer | 0.15 | 0.15 | 0.11 | 0.23 | 0.08 | 0.16 | 0.11 | 0.900 | 0.23 | 0.23 | 0.18 | 0.09 | 0.11 | 0.19 | 0.075 |
| C16 Cer | 2.03 | 1.88 | 2.86 | 2.42 | 1.76 | 2.33 | 2.13 | 0.759 | 3.49 | 4.12 | 2.79 | 1.48 | 1.94 | 2.02 | 0.017 |
| C18:1 Cer | 0.01 | 0.01 | 0.01 | 0.01 | 0.01 | 0.01 | 0.00 | 0.615 | 0.00 | 0.00 | 0.00 | 0.00 | 0.00 | 0.00 | N/A |
| C18:0 Cer | 0.15 | 0.19 | 0.24 | 0.17 | 0.19 | 0.22 | 0.20 | 0.949 | 0.19 | 0.13 | 0.12 | 0.05 | 0.09 | 0.06 | 0.046 |
| C20 Cer | 1.47 | 1.18 | 1.84 | 1.55 | 1.32 | 1.41 | 1.38 | 0.653 | 1.59 | 1.66 | 1.28 | 0.70 | 0.84 | 1.15 | 0.025 |
| C22 Cer | 16.58 | 15.37 | 14.75 | 18.36 | 11.37 | 10.07 | 13.95 | 0.379 | 50.22 | 56.08 | 44.47 | 19.92 | 35.61 | 30.65 | 0.020 |
| C24:1 Cer | 8.96 | 5.86 | 7.53 | 9.98 | 4.90 | 6.78 | 6.26 | 0.765 | 92.74 | 121.79 | 74.56 | 36.88 | 53.01 | 48.70 | 0.026 |
| C24:0 Cer | 14.14 | 15.78 | 12.93 | 20.37 | 10.27 | 11.49 | 13.49 | 0.896 | 96.54 | 97.09 | 83.09 | 42.04 | 64.61 | 53.76 | 0.008 |
| C26:1 Cer | 0.09 | 0.05 | 0.10 | 0.09 | 0.06 | 0.07 | 0.06 | 0.501 | 0.73 | 0.74 | 0.59 | 0.38 | 0.41 | 0.37 | 0.004 |
| C26:0 Cer | 0.14 | 0.10 | 0.22 | 0.22 | 0.10 | 0.19 | 0.18 | 0.643 | 0.28 | 0.38 | 0.35 | 0.31 | 0.22 | 0.24 | 0.121 |
|  | **d18 Sphingolipids Normalized values** | | | | | | | **t-test** | **d18 Sphingolipids Normalized values** | | | | | | **t-test** |
| **Species** | **Alb-Cre** | | | **SPT3-hKO** | | | | **KO vs WT** | **Alb-Cre** | | | **SPT3-hKO** | | | **KO vs WT** |
| d18:1 Sph | 43.23 | 40.06 | 40.36 | 58.61 | 63.11 | 59.47 | 51.66 | 0.002 | 69.61 | 45.09 | 69.70 | 51.86 | 48.29 | 33.68 | 0.164 |
| d18:0 DHS | 4.51 | 4.53 | 4.38 | 11.89 | 10.53 | 11.97 | 9.81 | 0.000 | 14.59 | 24.37 | 16.08 | 25.06 | 13.34 | 9.97 | 0.707 |
| d18:1 S1P | 3.54 | 3.01 | 3.73 | 1.61 | 1.19 | 1.22 | 0.95 | 0.000 | 2.33 | 3.46 | 3.79 | 1.69 | 2.66 | 2.67 | 0.195 |
| d18:0 DHS1P | 1.65 | 1.22 | 1.74 | 1.70 | 1.52 | 1.73 | 1.61 | 0.518 | 0.38 | 0.31 | 0.54 | 0.29 | 0.62 | 0.52 | 0.617 |
| C14 Cer | 0.20 | 0.21 | 0.16 | 0.18 | 0.27 | 0.19 | 0.17 | 0.700 | 0.24 | 0.19 | 0.19 | 0.08 | 0.11 | 0.12 | 0.007 |
| C16 Cer | 7.15 | 9.02 | 10.79 | 13.85 | 13.24 | 12.03 | 14.92 | 0.010 | 179.82 | 202.88 | 37.71 | 75.84 | 112.5 | 107.28 | 0.476 |
| C18:1 Cer | 0.15 | 0.12 | 0.14 | 0.13 | 0.10 | 0.11 | 0.09 | 0.065 | 0.18 | 0.26 | 0.16 | 0.15 | 0.11 | 0.09 | 0.067 |
| C18:0 Cer | 3.42 | 3.59 | 2.69 | 3.24 | 3.93 | 3.57 | 4.34 | 0.201 | 5.06 | 5.06 | 3.73 | 2.37 | 3.71 | 2.87 | 0.051 |
| C20 Cer | 88.4 | 81.55 | 88.77 | 103.7 | 106.9 | 95.68 | 112 | 0.010 | 135.39 | 129.76 | 100.33 | 49.78 | 83.46 | 67.86 | 0.020 |
| C22 Cer | 402.0 | 395.2 | 401.6 | 534.7 | 539.6 | 439.2 | 462.5 | 0.026 | 833.58 | 916.07 | 938.03 | 394.3 | 810.3 | 728.1 | 0.127 |
| C24:1 Cer | 330.1 | 320 | 317.7 | 443.4 | 353.2 | 388.7 | 372.1 | 0.035 | 1217.5 | 1429.1 | 1325.6 | 656.3 | 1082 | 963.9 | 0.040 |
| C24:0 Cer | 402.9 | 364.4 | 370.3 | 519.5 | 508.8 | 432.4 | 437.3 | 0.022 | 1365.2 | 1697.8 | 1288.3 | 688.1 | 1230 | 936.7 | 0.068 |
| C26:1 Cer | 1.40 | 1.33 | 1.90 | 1.74 | 1.29 | 1.69 | 1.32 | 0.883 | 6.17 | 8.59 | 6.23 | 4.11 | 4.27 | 3.51 | 0.022 |
| C26:0 Cer | 4.53 | 1.44 | 0.66 | 1.07 | 0.74 | 0.30 | 0.68 | 0.193 | 7.83 | 9.55 | 7.30 | 6.15 | 6.95 | 5.01 | 0.068 |
|  | **d19 Sphingolipids Normalized values** | | | | | | | **t-test** | **d19 Sphingolipids Normalized values** | | | | | | **t-test** |
| **Species** | **Alb-Cre** | | | **SPT3-hKO** | | | | **KO vs WT** | **Alb-Cre** | | | **SPT3-hKO** | | | **KO vs WT** |
| d19:1 Sph | 0.19 | 0.12 | 0.19 | 0.34 | 0.31 | 0.29 | 0.30 | 0.002 | 0.03 | 0.05 | 0.03 | 0.12 | 0.01 | 0.04 | 0.549 |
| d19:0 DHS | 2.45 | 2.55 | 4.29 | 2.13 | 1.76 | 1.29 | 0.97 | 0.045 | 0.00 | 0.00 | 0.00 | 0.00 | 0.00 | 0.00 | N/A |
| d19:1 S1P | 0.00 | 0.00 | 0.00 | 0.00 | 0.00 | 0.00 | 0.00 | N/A | 0.00 | 0.00 | 0.00 | 0.00 | 0.00 | 0.00 | N/A |
| d19:0 DHS1P | 0.00 | 0.00 | 0.00 | 0.00 | 0.00 | 0.00 | 0.00 | N/A | 0.00 | 0.00 | 0.00 | 0.00 | 0.00 | 0.00 | N/A |
| C14 Cer | 0.00 | 0.00 | 0.00 | 0.00 | 0.00 | 0.00 | 0.00 | 0.822 | 0.00 | 0.00 | 0.00 | 0.00 | 0.00 | 0.00 | N/A |
| C16 Cer | 0.06 | 0.09 | 0.09 | 0.10 | 0.10 | 0.09 | 0.15 | 0.151 | 0.10 | 0.07 | 0.07 | 0.03 | 0.04 | 0.03 | 0.007 |
| C18:1 Cer | 0.00 | 0.00 | 0.00 | 0.00 | 0.00 | 0.00 | 0.00 | N/A | 0.00 | 0.00 | 0.00 | 0.00 | 0.00 | 0.00 | N/A |
| C18:0 Cer | 0.06 | 0.05 | 0.09 | 0.07 | 0.11 | 0.06 | 0.13 | 0.292 | 0.09 | 0.05 | 0.05 | 0.04 | 0.03 | 0.04 | 0.133 |
| C20 Cer | 0.10 | 0.05 | 0.07 | 0.09 | 0.08 | 0.07 | 0.11 | 0.404 | 0.23 | 0.24 | 0.16 | 0.09 | 0.12 | 0.09 | 0.020 |
| C22 Cer | 1.11 | 0.78 | 1.01 | 1.59 | 1.00 | 0.73 | 1.40 | 0.422 | 2.33 | 3.38 | 3.10 | 1.47 | 2.53 | 2.01 | 0.100 |
| C24:1 Cer | 0.78 | 0.55 | 0.59 | 0.91 | 0.40 | 0.48 | 0.62 | 0.821 | 4.26 | 6.87 | 5.34 | 2.47 | 3.57 | 2.93 | 0.038 |
| C24:0 Cer | 0.59 | 0.41 | 0.55 | 1.02 | 0.60 | 0.73 | 0.62 | 0.128 | 2.27 | 3.19 | 2.77 | 2.10 | 2.51 | 1.91 | 0.147 |
| C26:1 Cer | 0.00 | 0.00 | 0.00 | 0.00 | 0.00 | 0.00 | 0.00 | N/A | 0.01 | 0.03 | 0.02 | 0.01 | 0.02 | 0.01 | 0.208 |
| C26:0 Cer | 0.00 | 0.00 | 0.00 | 0.00 | 0.00 | 0.00 | 0.00 | N/A | 0.02 | 0.02 | 0.01 | 0.02 | 0.02 | 0.01 | 0.810 |
|  | **d20 Sphingolipids Normalized values** | | | | | | | **t-test** | **d20 Sphingolipids Normalized values** | | | | | | **t-test** |
| **Species** | **Alb-Cre** | | | **SPT3-hKO** | | | | **KO vs WT** | **Alb-Cre** | | | **SPT3-hKO** | | | **KO vs WT** |
| d20:1 Sph | 0.16 | 0.22 | 0.21 | 1.27 | 0.87 | 0.62 | 0.57 | 0.021 | 0.35 | 0.10 | 0.16 | 0.30 | 0.17 | 0.17 | 0.926 |
| d20:0 DHS | 1.55 | 1.54 | 1.56 | 1.28 | 2.27 | 2.25 | 1.42 | 0.451 | 0.08 | 0.14 | 0.20 | 0.45 | 0.31 | 0.31 | 0.020 |
| d20:1 S1P | 0.13 | 0.13 | 0.14 | 0.08 | 0.09 | 0.11 | 0.10 | 0.001 | 0.03 | 0.03 | 0.05 | 0.02 | 0.02 | 0.02 | 0.173 |
| d20:0 DHS1P | 1.05 | 1.33 | 1.42 | 0.49 | 0.55 | 0.57 | 0.57 | 0.001 | 0.00 | 0.00 | 0.00 | 0.00 | 0.00 | 0.00 | N/A |
| C14 Cer | 0.00 | 0.00 | 0.00 | 0.00 | 0.00 | 0.00 | 0.00 | N/A | 0.00 | 0.00 | 0.00 | 0.00 | 0.00 | 0.00 | N/A |
| C16 Cer | 0.01 | 0.01 | 0.01 | 0.01 | 0.01 | 0.01 | 0.01 | 0.216 | 0.00 | 0.00 | 0.00 | 0.00 | 0.00 | 0.00 | N/A |
| C18:1 Cer | 0.00 | 0.00 | 0.00 | 0.00 | 0.00 | 0.00 | 0.00 | N/A | 0.00 | 0.00 | 0.00 | 0.00 | 0.00 | 0.00 | N/A |
| C18:0 Cer | 0.25 | 0.22 | 0.32 | 0.25 | 0.36 | 0.25 | 0.34 | 0.408 | 0.16 | 0.20 | 0.11 | 0.09 | 0.12 | 0.06 | 0.083 |
| C20 Cer | 0.10 | 0.10 | 0.16 | 0.19 | 0.16 | 0.19 | 0.21 | 0.020 | 0.10 | 0.13 | 0.10 | 0.08 | 0.12 | 0.09 | 0.399 |
| C22 Cer | 0.01 | 0.00 | 0.01 | 0.02 | 0.01 | 0.01 | 0.01 | 0.210 | 0.07 | 0.16 | 0.08 | 0.06 | 0.06 | 0.05 | 0.195 |
| C24:1 Cer | 0.01 | 0.01 | 0.01 | 0.00 | 0.00 | 0.00 | 0.01 | 0.008 | 0.02 | 0.05 | 0.02 | 0.03 | 0.02 | 0.01 | 0.506 |
| C24:0 Cer | 0.06 | 0.03 | 0.07 | 0.08 | 0.05 | 0.06 | 0.06 | 0.451 | 0.07 | 0.14 | 0.07 | 0.11 | 0.10 | 0.06 | 0.895 |
| C26:1 Cer | 0.01 | 0.01 | 0.01 | 0.00 | 0.00 | 0.01 | 0.00 | 0.142 | 0.00 | 0.00 | 0.01 | 0.00 | 0.00 | 0.00 | 0.431 |
| C26:0 Cer | 0.04 | 0.02 | 0.03 | 0.03 | 0.02 | 0.03 | 0.03 | 0.754 | 0.04 | 0.05 | 0.01 | 0.05 | 0.05 | 0.03 | 0.703 |
